# Oxidatively Locked [Co_2_L_3_]^6+^ Cylinders Derived from Bis(bidentate) 2-Pyridyl-1,2,3-triazole “Click” Ligands: Synthesis, Stability, and Antimicrobial Studies

**DOI:** 10.3390/molecules21111548

**Published:** 2016-11-16

**Authors:** Roan A. S. Vasdev, Dan Preston, Synøve Ø. Scottwell, Heather J. L. Brooks, James D. Crowley, Michael P. Schramm

**Affiliations:** 1 Department of Chemistry, University of Otago, P.O. Box 56, Dunedin 9016, Otago, New Zealand; roan.vasdev044@gmail.com (R.A.S.V.); preda458@student.otago.ac.nz (D.P.); synove.scottwell@unsw.edu.au (S.Ø.S.); 2 Department of Microbiology and Immunology, University of Otago, P.O. Box 56, 720 Cumberland Street, Dunedin 9054, Otago, New Zealand; heather.brooks@otago.ac.nz; 3 Department of Chemistry and Biochemistry, California State University, Long Beach, 1250 Bellflower Boulevard, Long Beach, CA 90840-9507, USA

**Keywords:** cobalt(III), metallosupramolecular, cylinders, antimicrobial agents

## Abstract

A small family of [Co_2_(**L_pytrz_**)_3_]^6+^ cylinders was synthesised from bis(bidentate) 2-pyridyl-1,2,3-triazole “click” ligands (**L_pytrz_**) through an “assembly-followed-by-oxidation” method. The cylinders were characterised using ^1^H, ^13^C, and DOSY NMR, IR, and UV-Vis spectroscopies, along with electrospray ionisation mass spectrometry (ESMS). Stability studies were conducted in dimethyl sulfoxide (DMSO) and D_2_O. In contrast to similar, previously studied, [Fe_2_(**L_pytrz_**)_3_]^4+^ helicates the more kinetically inert [Co_2_(**L_pytrz_**)_3_]^6+^ systems proved stable (over a period of days) when exposed to DMSO and were even more stable in D_2_O. The triply stranded [Co_2_(**L_pytrz_**)_3_]^6+^ systems and the corresponding “free” ligands were tested for antimicrobial activity in vitro against both Gram-positive (*Staphylococcus aureus*) and Gram-negative (*Escherichia coli*) microorganisms. Agar-based disk diffusion and Mueller–Hinton broth micro-dilution assays showed that the [Co_2_(**L_pytrz_**)_3_]^6+^ cylinders were not active against either strain of bacteria. It is presumed that a high charge of the [Co_2_(**L_pytrz_**)_3_]^6+^ cylinders is preventing them from crossing the bacterial cell membranes, rendering the compounds biologically inactive.

## 1. Introduction

There is emerging interest in the biological properties of metallosupramolecular architectures [1]. Building on the pioneering work of Lehn and co-workers [2] that demonstrated that cationic double-helical copper(I) complexes strongly interact with DNA [3], doubly, triply , and quadruply stranded helicate [4] architectures have all been found to display interesting biological behaviour. Hannon and co-workers discovered an inexpensive and simple methodology to synthesise a triply stranded [Ni_2_**L**_3_]^4+^ (**L** = (NE,N′E)-4,4′-methylenebis(*N*-(pyridin-2-ylmethylene)aniline)) helicate [4] and subsequently the related triply stranded [Fe_2_**L**_3_]^4+^ helicate. The latter displayed the ability to induce conformational changes in the DNA decamer [d(GACGGCCGTC)_2_] [5]. Hannon also showed that this triply stranded helicate non-covalently binds at the centre of three-way (Y-shaped) DNA [6,7,8,9] and RNA [10] junctions. The iron(II) [Fe_2_**L**_3_]^4+^ and ruthenium(II) [Ru_2_**L**_3_]^4+^ helicates have been found to display micromolar cytotoxicity against a range of cancer cell lines [11,12], while the [Fe_2_**L**_3_]^4+^ and [Ni_2_L_3_]^4+^ complexes inhibit Aβ amyloid aggregation. Additionally, the [Fe_2_**L**_3_]^4+^ helicate has been shown to display modest antibacterial activity against *Bacillus subtilis* (minimum inhibitory concentration (MIC) = 32 µg·mL^−1^) and *Escherichia coli* (MIC = 64 µg·mL^−1^) [13].

More recently, Scott and co-workers developed a family of related water-stable, zinc(II), and iron(II) pyridylimine “flexicates” that contain a range of more flexible spacer units between the two pyridylimine coordination sites. Like the system of Hannon and coworkers, these flexicates were shown to be potent (IC_50_ = 40 nM) anticancer agents [14,15,16] and inhibit Aβ amyloid aggregation [17]. Some of these flexicates also displayed good antimicrobial activity against both Gram-positive *Staphylococcus aureus* (8 < MIC > 128 μg·mL^−1^) and Gram-negative *E. coli* (4 < MIC > 128 μg·mL^−1^) bacteria [18]. 

**Scheme 1:**
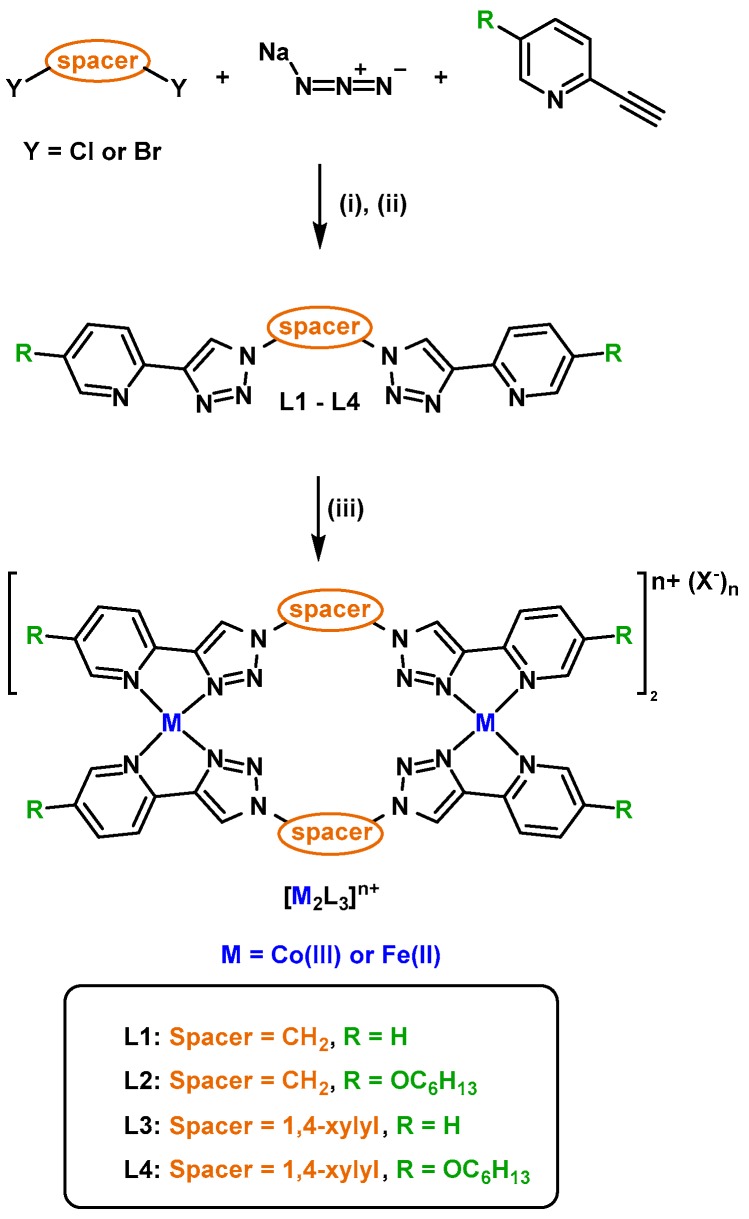
Synthesis of ligands (**top**) and cylinders (**bottom**): (i) DMF/H_2_O (4:1) microwave 110 °C, 125 W; 2 h; (ii) CuSO_4_·5H_2_O, sodium ascorbate, RT, 14 h; (iii) (a) [Fe(OH_2_)_6_](BF_4_)_2_, CH_3_CN, RT, 5 min or (b) [Co(OH_2_)_6_](BF_4_)_2_·CH_3_CN, RT, 5 min then cerium ammonium nitrate, CH_3_CN, RT, 5 min then NH_4_PF_6_, DMSO, and H_2_O, RT, 5 min or (c) [Co(OH_2_)_6_](BF_4_)_2_, CH_3_CN, RT, 5 min, then cerium ammonium nitrate, CH_3_CN, RT, 5 min, then tetrabutylammonium triflate, DMSO, RT, 5 min.

The copper(I)-catalysed 1,3-dipolar cycloaddition of azides and terminal alkynes to regioselectively produce 1,4-disubstituted-1,2,3-triazoles (CuAAC “click” reaction) [19,20] has become a versatile synthetic tool for the construction of functional ligand architectures [21,22,23,24,25]. These “click” ligands have been exploited for a range of purposes including the construction of metallosupramolecular macrocycles [26,27,28,29] and helicates [30,31]. In a previous work, we have exploited “click” ligands to synthesise triply [32,33] and quadruply [34] stranded helicates and explored the biological activity of these systems. We showed that a dipalladium quadruply stranded helicate had good cytotoxicity (IC_50_ = 3–7 µM) against three different cancer cell lines; however, disappointingly, the complex displayed no selectivity towards non-cancerous phenotypes. Inspired by the iron(II) systems developed by Hannon and Scott, we used **L3** (Scheme 1) and some other related bis(bidentate) 2-pyridyl-1,2,3-triazole “click” ligands (**L_pytrz_**) to synthesise a small family of iron(II) cylinders [Fe_2_(**L_pytrz_**)_3_]^4+^ [32]. Unfortunately, these [Fe_2_(**L_pytrz_**)_3_]^4+^ cylinders displayed no biological activity (against the yeast *Saccharomyces cerevisiae*). The lack of activity was traced back to the lability of these systems, the [Fe_2_(**L_pytrz_**)_3_]^4+^ cylinders rapidly decomposed in the presence of dimethylsulfoxide (DMSO) and biological nucleophiles. In an effort to generate more biologically robust cylinders, we synthesised the ruthenium(II) triply stranded helicate [Ru_2_(**L3**)_3_]^4+^ [33]. This more kinetically inert system proved more stable in the presence of DMSO and biological nucleophiles and was found to display very moderate antimicrobial activity (MIC > 256 μg·mL^−1^) against both *S. aureus* and *E. coli* bacteria strains [33]. Unfortunately, efforts to extend the family of ruthenium(II) cylinders with other **L_pytrz_** ligands were unsuccessful. Only polymeric complexes were obtained due to the more inert nature of the ruthenium(II) preventing the error-correction process required for clean self-assembly of the metallosupramolecular complexes.

Very recently, Lusby and co-workers reported the synthesis of some kinetically “locked” cobalt(III) tetrahedral cages, which were generated through an “assembly-followed-by-oxidation” process [35,36]. Taking advantage of the different labilities of Co(II) and Co(III), the tetrahedral cages were assembled under thermodynamic control and then “locked” upon oxidation of the Co(II) ions to Co(III). We reasoned that this approach may enable the generation of a family of biologically robust and active [Co_2_(**L_pytrz_**)_3_]^6+^ cylinders. Cobalt is a physiologically active metal that has key functions in many biological processes [37]. Because of this, more and more cobalt complexes are being explored for an extensive range of biological applications such as anti-inflammatory [38], antiviral [39], anticancer [40], and antimicrobial [41,42] agents amongst others [43]. Consequently, development of cobalt complexes could significantly enhance the arsenal of pharmaceutical drugs available to combat a wide variety of harmful diseases and reduce antibiotic resistance.

Herein, we report the synthesis and characterisation of a small series of [Co_2_(**L_pytrz_**)_3_](OTf)_6_ cylinders assembled using bis(2-pyridyl-1,2,3-triazole) ligands. Exploiting the “assembly-followed-by-oxidation” process, labile Co(II) ions were used to form helicates, and oxidation to Co(III) yielded the kinetically inert complexes. Additionally, we examined the stability of the [Co_2_ (**L_pytrz_**)_3_]^6+^ cylinders in a range of solvents and in the presence of biologically relevant nucleophiles along with their antimicrobial activity against *S. aureus* and *E. coli*.

## 2. Results

### 2.1. Cylinder Design and Synthesis

The target 2-pyridyl-1,2,3-triazole ligands (**L1**–**L4**) consist of 1,2,3-triazoles linked with either a methylene (**L1**, **L2**) or a 1,4-xylyl (**L3**, **L4**) unit, with a terminal pyridine either unsubstituted (**L1**, **L3**) or decorated with a hexyloxy moiety (**L2**, **L4**) (Scheme 1). The ligands (**L1** [26], **L2** [26], and **L3** [44]) were constructed in good yields using previously reported methods. The new ligand **L4** was synthesised with a 72% yield using an analogous procedure and characterised via ^1^H-NMR, ^13^C-NMR, and infrared spectroscopies and high-resolution electrospray mass spectrometry (HR-ESMS) (Scheme 1, Supplementary Materials, Figures S1 and S2).

**Figure 1:**
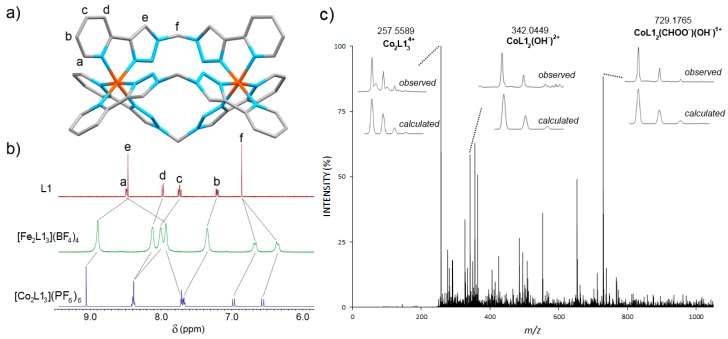
(**a**) Crystal structure of [Fe_2_**L1**_3_](BF_4_)_4_ (hydrogen atoms, solvent molecules, and counter-ions have been omitted for clarity) Colours: carbon = grey; nitrogen = blue; iron = orange; (**b**) partial stacked ^1^H-NMR spectra (400 MHz, 298 K, CD_3_CN) of **L1**, [Fe_2_**L1**_3_](BF_4_)_4_ and [Co_2_**L1**_3_](PF_6_)_6_; (**c**) representative mass spectrum of [Co_2_**L1**_3_](OTf)_6_.

With the ligands in hand, the Co(III) cylinders were generated using an “assembly-followed-by-oxidation” approach, labile Co(II) salts were used to form helicates, then oxidation to Co(III) yielded the kinetically inert cylinders. Whilst oxidation of Co(II) to Co(III) via bromine [45], air [46], and chloranil [47] has previously been reported, we exploited the excellent methodology of Lusby and co-workers: using cerium ammonium nitrate as an oxidant [35,36]. Complexation of one of the ligands (**L1**–**L4**) with [Co(OH_2_)_6_](BF_4_)_2_ in a 3:2 ratio yielded Co(II) complexes. In situ ^1^H-NMR spectroscopy (CD_3_CN, 298 K) indicated that the [Co_2_(**L_pytrz_**)_3_]^4+^ cylinders formed. No “free” ligand could be detected, and all of the proton signals were paramagnetically broadened and shifted over a wide chemical shift range (0–100 ppm, Supplementary Materials, Figure S3) as has been observed previously [35,36,48,49,50]. We did not attempt to isolate the cobalt(II) [Co_2_(**L_pytrz_**)_3_]^4+^ cylinders, they were oxidised to the corresponding [Co_2_(**L_pytrz_**)_3_]^6+^ cylinders using [Ce(NO_3_)_6_](NH_4_)_2_. Tetrabutylammonium triflate or saturated aqueous ammonium hexafluorophosphate was then added in a 30-fold excess before precipitation provided the [Co_2_(**L_pytrz_**)_3_](X)_6_ (where X = PF_6_^−^ or OTf^−^) cylinders in good yields (69%–84%) as yellow-orange powders. The formation of the [Co_2_(**L_pytrz_**)_3_]^6+^ cylinders was confirmed via ^1^H-NMR, ^13^C-NMR, infrared, and UV-Vis spectroscopies and high-resolution electrospray mass spectrometry (Supplementary Materials, Figures S4–S26). ^1^H-NMR spectra of the complexes show downfield shifts relative to the free ligands of the pyridyl (H_b–d_) and triazole (H_e_) proton resonances consistent with complexation to the cobalt(III) ions (Figure 1b, Supplementary Materials, Figures S4–S26). Additionally, the methylene (CH_2_) protons of the linkers are split into a characteristic AB quartet, indicative of the ligand being conformationally locked upon formation of the complexes, similar to what has been observed for other **L_pytrz_**-derived cylinders [30,31,32,33]. ^1^H-NMR DOSY spectra (*d*_6_-DMSO) were also consistent with the formation of the [Co_2_(**L_pytrz_**)_3_](OTf)_6_ complexes (Supplementary Materials, Figure S27, Table S1). The diffusion coefficients (D) range from 1.28–0.96 × 10^−10^ m^2^·s^−1^, values that are similar to those observed for the related [Ru_2_(**L1**)_3_]^4+^ complex in *d*_6_-DMSO (Supplementary Materials, Table S1). Furthermore, the log of diffusion coefficients of the ligands (**L1**–**L4**) and the corresponding [Co_2_(**L_pytrz_**)_3_](OTf)_6_ complexes along with the values for two related crystallographically characterised complexes—[Ru_2_**L3**_3_](PF_6_)_4_ and [Pt**L1**_2_](PF_6_)_2_—were plotted against the log of the molecular weight (M_w_) of the compounds (Supplementary Materials, Figure S27). This plot showed a strong linear correlation (R^2^ = 0.962) between the log(D) and log(M_w_) of the compounds providing additional support for the formation of the [Co_2_(**L_pytrz_**)_3_](OTf)_6_ cylinders. The ESMS mass spectra of [Co_2_(**L_pytrz_**)_3_](OTf)_6_ (Figure 1c, Supplementary Materials, Figures S9, S15, S20, and S25) were also consistent with the formation of the expected cylinders. Isotopically resolved peaks consistent with the presence of [Co_2_(**L_pytrz_**)_3_]^4+^ ions along with other ions due to fragmentation were observed for each cobalt complex.

Unfortunately, despite extensive efforts, crystals of the cobalt(III) cylinders of the quality required for X-ray diffraction (XRD) studies could not be obtained [51]. The analogous [Fe_2_**L3**_3_](BF_4_)_4_, and [Ru_2_**L3**_3_](PF_6_)_4_ cylinders have been previously crystallographically characterised and were found, in both cases, to be racemic mixtures of the ΔΔ and ΛΛ helicates [30,32,33]. In order to gain some structural information on the cobalt(III) cylinders derived from **L1** and **L2**, we synthesised the related iron(II) complex [Fe_2_**L1**_3_](BF_4_)_4_ (Supplementary Materials, Figures S28 and S29). Crystals suitable for XRD studies of [Fe_2_**L1**_3_](BF_4_)_4_ were obtained via vapor diffusion of diethyl ether into acetonitrile. The complex crystallised in the *P*$\bar{1}$ space group. The asymmetric unit contained the expected iron(II) metallosupramolecular cylinder [Fe_2_**L1**_3_]^4+^, 4 tetrafluoroborate anions, and 4.4CH_3_CN solvent molecules (Figure 1a, Supplementary Materials, Table S2 and Figure S32). Unlike the [Fe_2_**L3**_3_](BF_4_)_4_ and [Ru_2_**L3**_3_](PF_6_)_4_ systems, which were helicates, the [Fe_2_**L1**_3_]^4+^ cylinder was found to be a mesocate with one tris(bidentate)iron(II) centre displaying the Δ configuration, and the other the Λ. The formation of the mesocate architecture with **L1** is consistent with the odd-even rule [52], which states that bis(bidentate) ligands with odd numbers of carbons in the spacer unit will form mesocates while ligands with even numbers of carbons in the spacer unit will generate helicates. This behaviour has been previously observed for [M_2_(**L_pytrz_**)_3_]^n+^ cylinders [30,31,32,33]. The ^1^H-NMR spectra (CD_3_CN, 298 K) of the crystallographically characterised [Fe_2_**L1**_3_](BF_4_)_4_ and [Fe_2_**L3**_3_](BF_4_)_4_ were compared to the analogous [Co_2_**L1**_3_](PF_6_)_6_ and [Co_2_**L3**_3_](PF_6_)_6_ (Figure 1b and Supplementary Materials, Figure S30). Overall, the data were very similar with the spectra of the cobalt(III) systems sharper and the proton resonances shifted slightly further downfield relative to the iron(II) complexes. This is presumably because the cobalt ions have a higher 3+ charge and are more inert. However, the spectral similarity to the related iron(II) systems suggests that the [Co_2_**L1**_3_]^6+^ and [Co_2_**L2**_3_]^6+^ adopt a mesocate structure in solution while the [Co_2_**L3**_3_]^6+^ and [Co_2_**L4**_3_]^6+^ are likely racemic mixtures of the ΔΔ and ΛΛ helicates.

### 2.2. Stability Studies

As previous work had shown that the [Fe_2_(**L_pytrz_**)_3_](BF_4_)_4_ cylinders were unstable in the presence of DMSO [32], we examined the stability of the new [Co_2_(**L_pytrz_**)_3_](OTf)_6_ cylinders under a variety of conditions before carrying out antimicrobial testing. To allow a direct comparison to the iron(II) systems that were examined previously, we initially studied the stability of the [Co_2_(**L_pytrz_**)_3_](OTf)_6_ cylinders in *d*_6_-DMSO in the light. The cylinders were dissolved in *d*_6_-DMSO and ^1^H-NMR spectra were obtained over a period of days (Supplementary Materials, Section 3). Like the related iron(II) cylinders, the cobalt(III) systems decompose in *d*_6_-DMSO. However, due to the more inert nature of d^6^ cobalt(III) ions relative to iron(II), the cylinders are observed to decompose over a period of days rather than instantaneously. Interestingly, the family of cobalt cylinders displayed different rates of decomposition. After two days, the [Co_2_**L1**_3_]^6+^ complex is completely decomposed into “free” **L1** and presumably [Co(*d*_6_-DMSO)_6_]^3+^. The [Co_2_**L2**_3_]^6+^ complex is more long lived and complete conversion to free ligand is only observed after four days. The larger [Co_2_**L3**_3_]^6+^ and [Co_2_**L4**_3_]^6+^ are more stable again. The [Co_2_**L3**_3_]^6+^ completely decomposed into “free” **L3** after six days and the [Co_2_**L4**_3_]^6+^ complex is still the major species (>70%) in solution after the same time period. The differences in the rates of decomposition can be attributed to a number of factors. The hexyloxy substituted ligands (**L2** and **L4**) are more electron rich relative to **L1** and **L3** leading to more stable complexes. The greater stability of the larger [Co_2_**L3**_3_]^6+^ and [Co_2_**L4**_3_]^6+^ complexes is presumably due to the lower charge repulsion between the more distant Co(III) cations and the lessening of the lone pair-lone pair repulsion that is present in the smaller [Co_2_**L1**_3_]^6+^ and [Co_2_**L2**_3_]^6+^ compounds (Supplementary Materials, Figure S48).

**Figure 2:**
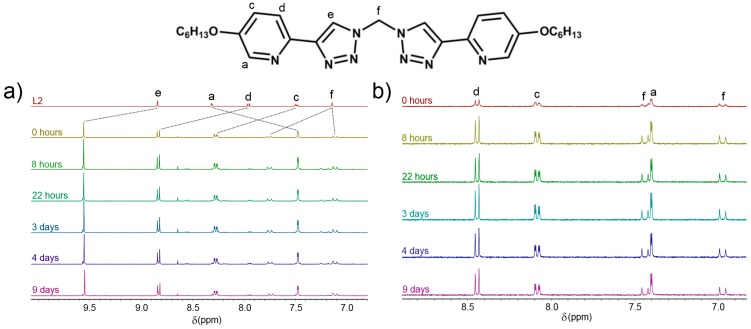
Partial stacked ^1^H-NMR spectra (400 MHz, 298 K) of (**a**) [Co_2_**L2**_3_](OTf)_6_ in *d*_6_-DMSO in the dark and (**b**) [Co_2_**L2**_3_](OTf)_6_ in D_2_O in the light. (Note: H_d_ is not observed in the D_2_O spectrum due to hydrogen-deuterium exchange, and the ligands are not soluble in D_2_O).

Intriguingly, repeating the *d*_6_-DMSO stability experiments in the absence of light resulted in a different outcome. The cobalt(III) cylinders are all much more stable in *d*_6_-DMSO when light is excluded from the reaction mixture. After six days in the dark, only very small peaks consistent with the “free” ligands can be observed in each of the examined systems (Figure 2a and Supplementary Materials, Section 3). The major species present in solution are the intact [Co_2_(**L_pytrz_**)_3_](OTf)_6_ cylinders. This suggests that the decomposition of the complexes in *d*_6_-DMSO is a light activated process, possibly due to in situ photoreduction of the cobalt(III) ions to the more labile cobalt(II) ions.

As the [Co_2_(**L_pytrz_**)_3_](OTf)_6_ cylinders were soluble in water, we have undertaken similar stability studies in D_2_O in the presence of light (Figure 2b and Supplementary Materials, Section 3). The stability of the complexes in D_2_O was significantly better than that observed in *d*_6_-DMSO in the presence of light. The cobalt(III) complexes showed no signs of decomposition over a period of a week in D_2_O, and no new signals are observed in any of the ^1^H-NMR spectra. Furthermore, no signals due to “free” ligands are observed, and no precipitate is formed during the monitored time period. In some cases, the acidic triazole and methylene protons of the complexes slowly disappear due to hydrogen-deuterium exchange, but the remaining proton resonances do not shift, confirming that the cylinders remain intact in D_2_O.

**Figure 3:**
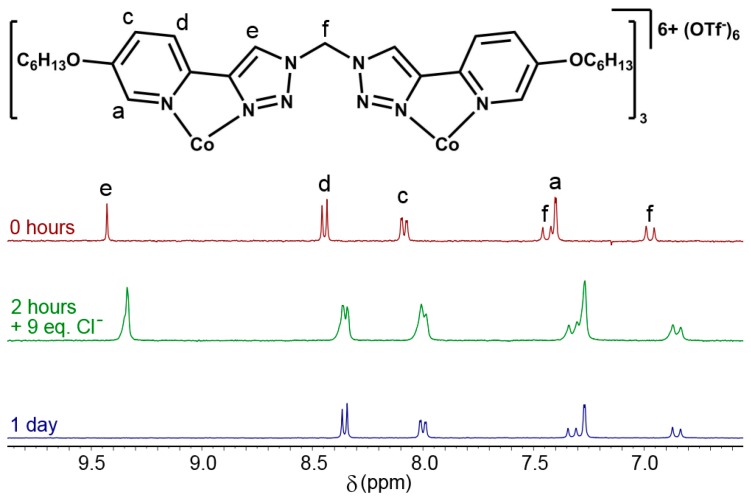
Partial stacked ^1^H-NMR (400 MHz, 298 K, D_2_O) of [Co_2_**L2**_3_](OTf)_6_ in the presence of Cl^−^ anions.

Additional stability tests, in D_2_O, were also conducted with the [Co_2_**L2**_3_](OTf)_6_ cylinder against the common biological nucleophiles histidine and chloride ions. [Co_2_**L2**_3_](OTf)_6_ (1 equiv) and tetrabutylammonium chloride (9 equiv) were dissolved in D_2_O, and the mixture was analysed via ^1^H-NMR spectroscopy (Figure 3). The complex proved stable over a day; as seen below, the acidic triazole (H_d_) slowly disappears due to hydrogen-deuterium exchange and the slight upfield shifts of all signals due to the increased concentration of negatively charged ions in solution. However, the characteristic AB quartet remains unchanged, confirming the stability of the cylinder under these conditions. ^1^H-NMR spectroscopy (D_2_O) on a mixture of [Co_2_**L2**_3_](OTf)_6_ (1 equiv) and histidine (6 equiv) provided similar results. While hydrogen-deuterium exchange was observed for the protons H_e_ and H_f_ of the complexes, the pyridyl resonances (H_a–d_) were unchanged over two days, and no sign of ligand precipitation from the solution was observed, indicating that the cylinder remained intact (Supplementary Materials, Section 3, Figure S47).

### 2.3. Antimicrobial Activity

The resistance of bacteria towards antibiotics is fast becoming a global issue. With pathogens developing a resistance to beta-lactams and other commonly used antibiotics, the need for new antimicrobial medicines is growing. Metal-containing antibacterial drugs have become a global research focus over the past decade and are showing increasing promise [53,54,55]. As the previously investigated, biologically robust, ruthenium(II) triply stranded helicate [Ru_2_(**L3**)_3_]^4+^ and related dirhenium(I) **L_pytrz_** complexes had displayed modest antimicrobial activity [33,56], we screened the new [Co_2_(**L_pytrz_**)_3_](OTf)_6_ cylinders against *S. aureus* (Gram-positive bacteria) and *E. coli* (Gram-negative bacteria). The activity of the ligands (**L1**–**L4**) and the cobalt(III) complexes was initially examined using Kirby–Bauer disk diffusion assays, but none of the ligands or cylinders displayed any zones of inhibition, indicating that they were not biologically active. As this negative result could potentially be due to the solubility properties of the compounds preventing the diffusion of materials from the disk into the agar, we further investigated the antimicrobial properties using Mueller–Hinton broth micro-dilution assays. Unfortunately, these experiments also indicated that the cobalt(III) cylinders are not active antimicrobial agents, and no inhibition of bacterial growth was observed over a range of concentrations, from 1024 μg·mL^−1^ to 1 μg·mL^−1^. Given the stability of the [Co_2_(**L_pytrz_**)_3_](OTf)_6_ cylinders in aqueous and pseudo-biological conditions and the activity of other triply stranded helicates [13,18], related dinuclear ruthenium(II) compounds [53,57], and cobalt(III) complexes [41,42], the observed lack of activity was initially surprising. However, a series of elegant papers from Keene and co-workers have shown that the charge of the metal complex is an important factor for obtaining active antimicrobial complexes. They have developed a family of highly active dinuclear ruthenium(II) complexes that are overall 4+ cations [53,57]. Subsequently, they generated the analogous dinuclear iridium(III) complexes that are overall 6+ cations and found that these compounds displayed no antimicrobial activity [58]. The have also investigated the corresponding monometallic systems [Ir(Me_4_phen)_3_]^3+^ and [Ru(Me_4_phen)_3_]^3+^ (where Me_4_phen = 3,4,7,8-tetramethyl-1,10-phenanthroline) and shown while the 2+ ruthenium system is highly active the 3+ iridium is not [59]. Based on these findings, Keene and co-workers have suggested that an individual metal centre with a 3+ charge cannot readily pass across the bacterial cell membrane, and our observed results are consistent with that postulate. 

## 3. Conclusions

A small family of the [Co_2_(**L_pytrz_**)_3_](OTf)_6_ cylinders were synthesised, in good yields, from bis(bidentate) 2-pyridyl-1,2,3-triazole “click” ligands through an “assembly-followed-by-oxidation” method. The cylinders were characterised using ^1^H, ^13^C, and DOSY NMR, IR, and UV-Vis spectroscopies, along with electrospray ionisation mass spectrometry (ESMS). Stability studies were conducted in DMSO and D_2_O in the presence and absence of light. In contrast to similar, previously studied, [Fe_2_(**L_pytrz_**)_3_]^4+^ helicates, the more kinetically inert [Co_2_(**L_pytrz_**)_3_]^6+^ systems proved stable (over a period of days) when exposed to dimethyl sulfoxide (DMSO). The cobalt(III) cylinders displayed impressive stability in D_2_O and retained their structure, even in the presence of common biological nucleophiles. In vitro agar-based disk diffusion and Mueller–Hinton broth assays with both *S. aureus* and *E. coli* showed that the [Co_2_**L**_3_]^6+^ cylinders were not biologically active against either strain of bacteria. It is presumed that the lack of biological activity is connected to the high charge of the [Co_2_**L**_3_]^6+^ cylinders, which is preventing them from crossing the bacterial cell membranes. These results suggest that, while the “assembly-followed-by-oxidation” approach would allow the generation of a large family of [Co_2_(**L_pytrz_**)_3_](OTf)_6_ cylinders that are kinetically robust under biological conditions, the high 6+ charge of the complexes will prevent their use as antimicrobial agents. 

## 4. Experimental

### 4.1. General

Unless otherwise stated, all reagents were purchased from commercial sources and used without further purification. The ligands **L1** [26], **L2** [26] and **L3** [44] were synthesised using our previously reported procedures. Solvents were laboratory reagent grade. Petroleum ether refers to the fraction of petrol boiling in the range 40–60 °C. Substances (and abbreviations) used in this study include isopropyl alcohol (IPA), methanol (CH_3_OH), dichloromethane (CH_2_Cl_2_), ethylenediaminetetraacetate (EDTA), tetrahydrofuran (THF), dimethyl sulfoxide (DMSO), and dimethylformamide (DMF). ^1^H-, ^13^C- and ^19^F-NMR spectra were recorded on either a 400 MHz Varian 400-MR or a 500 MHz Varian AR spectrometer. Chemical shifts are reported in parts per million and referenced to residual solvent peaks (CDCl_3_: ^1^H δ 7.26 ppm, ^13^C δ 77.16 ppm; CD_3_CN: ^1^H δ 1.94, ^13^C δ 1.32, 118.26 ppm, *d*_6_-DMSO: ^1^H δ 2.50 ppm; ^13^C δ 39.52 ppm; CD_3_NO_2_: ^1^H δ 4.33, ^13^C δ 57.3 ppm). Coupling constants (*J*) are reported in Hertz (Hz). Standard abbreviations indicating multiplicity were used as follows: m = multiplet, t = triplet, q = quartet, quin = quintet, dt = double triplet, d = doublet, dd = double doublet, s = singlet, and br = broad. IR spectra were recorded on a Bruker ALPHA FT-IR spectrometer with an attached ALPHA-P measurement module. A CEM S-class microwave reactor was used to carry out microwave enhanced reactions. Microanalyses were performed at the Campbell Microanalytical Laboratory at the University of Otago. Electrospray mass spectra (ESMS) were collected on a Bruker micrOTOF-Q spectrometer. UV-Vis spectra were recorded on a Perkin Elmer Lambda 950 UV-Vis/NIR spectrometer.

**Synthesis of L4:** A mixture of 1,4-bis(bromomethyl)benzene (96 mg, 0.363 mmol) and NaN_3_ (47.2 mg, 0.726 mmol) in DMF/water (10 mL, 4:1) was heated in a CEM microwave reactor (250 W) for 1.5 h at 125 °C then allowed to cool to room temperature. After an addition of 5-(hexyloxy)-2-((trimethylsilyl)ethynyl)pyridine (200 mg, 0.726 mmol), CuSO_4_·5H_2_O (45.3 mg, 0.182 mmol), sodium ascorbate (72 mg, 0.363 mmol), and Na_2_CO_3_ (154 mg, 1.452 mmol), the reaction mixture was stirred at room temperature for 16 hours. It was then added to a 0.1 M EDTA/NH_4_OH aqueous solution (100 mL) and to CH_2_Cl_2_ (100 mL) and stirred vigorously for one hour. The organic layer was then washed with water (5 × 100 mL) and brine (100 mL), dried over Na_2_SO_4_, and filtered, and the solvent was removed under vacuum. Column chromatography (silica gel) (CH_2_Cl_2_ then 4:1 CH_2_Cl_2_/acetone) afforded the product as a fluffy white solid (155 mg, 0.260 mmol, 72%). ^1^H-NMR (500 MHz, CDCl_3_, 298 K) δ: 8.22 (d, *J* = 2.9 Hz, 2H, H_a_), 8.08 (d, *J* = 8.7 Hz, 2H, H_d_), 7.94 (s, 2H, H_e_), 7.33 (s, 4H, H_g_), 7.25 (dd, *J* = 2.9, 9.0 Hz, 2H, H_c_ (under residual solvent peak)), 5.56 (s, 4H, H_f_), 4.01 (t, *J* = 6.5 Hz, 4H, H_h_), 1.85–1.75 (m, 4H, H_i_), 1.48 (quin, *J* = 7.2 Hz, 4H, H_j_), 1.38–1.31 (m, 8H, H_k,l_), 0.91 (t, *J* = 7.0 Hz, 6H, H_m_). Diffusion coefficient (*d*_6_-DMSO, 298 K) D: 1.86 × 10^−10^ m^2^·s^−1^. ^13^C-NMR (125 MHz, CDCl_3_, 298 K) δ: 155.3, 135.3, 132.3, 132.2, 129.1, 128.7, 128.6, 122.3, 121.7, 69.1, 54.0, 31.6, 29.1, 25.7, 22.7, 14.1. ATR-IR: ν (cm^−1^) 3118, 2933, 1577, 1470, 1384, 1275, 1230, 1045, 849. HR-ESMS (CH_3_OH) *m*/*z* = 617.3291 [M + Na]^+^ (calc. for C_34_H_42_N_8_NaO_2_, 617.3323). Anal. calcd. for C_34_H_42_N_8_O_2_∙0.8H_2_O: C, 67.04; H, 7.21; N, 18.39%. Found: C, 67.03; H, 7.10; N, 18.12%.

**Synthesis of [Co_2_L1_3_](PF_6_)_6_:** A solution of [Co(H_2_O)_6_](BF_4_)_2_ (31 mg, 91 µmol) in acetonitrile (0.055 mL) was added to a suspension of **L1** (41 mg, 137 µmol) in acetonitrile (1 mL). An addition of a solution of (NH_4_)_2_[Ce(NO_3_)_6_] (75 mg, 137 µmol) in acetonitrile (0.440 mL) to this solution resulted in the precipitation of a fluffy yellow solid. After centrifugation (13,000 RPM, 5 min), the filtrate was discarded, and the pellet was suspended in acetonitrile and collected by filtration. The solid obtained was dissolved in a minimal amount of water before an addition of a saturated aqueous solution of [NH_4_]PF_6_ (10 mL). The resulting precipitate was collected by filtration, washed with water (2 × 5 mL), dissolved in acetonitrile (5 mL), and filtered through cotton wool. Vapor diffusion of diethyl ether into this solution yielded the product as yellow crystals (38 mg, 27 µmol, 60%). ^1^H-NMR (500 MHz, *d*_6_-DMSO, 298 K) δ: 9.71 (s, 6H, H_e_), 8.89 (dd, *J* = 7.9, 1.4 Hz, 6H, H_d_), 8.64 (t, *J* = 7.7 Hz, 6H, H_c_), 7.97 (d, *J* = 6.0 Hz, 6H, H_a_), 7.89 (dt, *J* = 6.8, 1.5 Hz, 6H, H_b_), 7.67 & 7.00 (ABq, *J*_AB_ = 14.2 Hz, 6H, H_f_). ^13^C-NMR (125 MHz, CD_3_CN, 298 K) δ: 153.9, 148.9, 148.4, 145.4, 131.0, 130.1, 127.0, 64.1. HR-ESMS (CH_3_CN) *m*/*z* = 805.0449 [M − 2PF_6_]^2+^ (calc. for C_45_H_36_Co_2_F_24_N_24_P_4_, 805.0387), 488.3749 [M − 3PF_6_]^3+^ (calc. for C_45_H_36_Co_2_F_18_N_24_P_3_, 488.3709). Anal. calcd. for C_45_H_36_Co_2_F_36_N_24_P_6_: C, 28.44; H, 1.91; N, 17.69%. Found: C, 28.49; H, 2.15; N, 17.44%.

### 4.2. General Synthetic Procedure for *[Co_2_**L_3_**](OTf)_6_* Compounds

**Synthesis of [Co_2_L1_3_](OTf)_6_:** A solution of [Co(OH_2_)_6_](BF_4_)_2_ (2 equiv) in CH_3_CN (2 mL) was added to a suspension of **L** (3 equiv) in CH_3_CN (2 mL). To this, a solution of (NH_4_)_2_[Ce(NO_3_)_6_] (2.2 equiv) in CH_3_CN (2 mL) was added to form a yellow precipitate. The solid was collected via centrifugation (5 min at 13,000 rpm) and dissolved in DMSO (0.75 mL). [NBu_4_](OTf) (30 equiv) was added as a solid to the orange solution. The product was collected by precipitation out of DMSO with ethyl acetate (3 mL) and diethyl ether (5 mL) then dried under vacuum.

**Synthesis of [Co_2_L1_3_](OTf)_6_:** [Co(OH_2_)_6_](BF_4_)_2_: 41 mg, 0.120 mmol, **L1**: 55 mg, 0.181 mmol, [Ce(NO_3_)_6_](NH_4_)_2_: 73 mg, 0.132 mmol, [N(C_4_H_9_)_4_](OTf): 707 mg, 1.806 mmol. Yellow solid (60 mg, 31 μmol, 75%) ^1^H-NMR (500 MHz, CD_3_NO_2_, 298 K) δ: 9.81 (s, 1H, H_e_), 8.59–8.47 (m, 2H, H_c,d_), 8.03 (d, *J* = 6.0 Hz, 1H, H_b_), 7.83 (ddd, *J* = 7.6, 6.0, 1.9 Hz, 1H, H_a_), 7.68 & 6.86 (ABq, *J*_AB_ = 14.1 Hz, 6H, H_f_). Diffusion coefficient (*d*_6_-DMSO, 298 K) D: 1.28 × 10^−10^ m^2^·s^−1^. ^13^C-NMR (125 MHz, CD_3_NO_2_, 298 K) δ: 148.3, 144.3, 143.8, 140.4, 125.9, 125.6, 122.0, 59.2. ^19^F-NMR (376 MHz, D_2_O, 298 K) δ: −78.9 (s). ATR-IR: ν (cm^−1^) 3106, 1587, 1338, 1253, 1028, 776, 636. HR ESI-MS (DMSO/CH_3_CN) *m/z* = 729.1765 [M − Co − L1 − 6OTf + CHOO + OH]^+^ (calc. for C_31_H_26_CoN_16_O_3_, 729.1700), 342.0449 [M − Co − L1 − 6OTf + OH]^2+^ (calc. for C_30_H_25_CoN_16_O, 342.0589), 257.5589 [M − 6OTf]^4+^ (calc. for C_45_H_36_Co_2_N_24_, 257.5549). UV-Vis (DMF, ε [M^−1^·cm^−1^]): λ_max_ nm = 442 (1.95 × 10^4^).

**Synthesis of [Co_2_L2_3_](OTf)_6_:** [Co(OH_2_)_6_](BF_4_)_2_: 28 mg, 0.083 mmol, **L2**: 63 mg, 0.124 mmol, (NH_4_)_2_[Ce(NO_3_)_6_]: 50 mg, 0.091 mmol, [NBu_4_](OTf): 487 mg, 1.244 mmol. Yellow solid (62 mg, 25 μmol, 78%) ^1^H-NMR (500 MHz, CD_3_NO_2_, 298 K) δ: 9.80 (s, 6H, H_d_), 8.50 (d, *J* = 8.9 Hz, 6H, H_c_), 8.02 (dd, *J* = 9.0, 2.5 Hz, 6H, H_c_), 7.84 & 6.95 (ABq, *J*_AB_ = 14.1 Hz, 6H, H_f_), 7.67 (d, *J* = 2.5 Hz, 6H, H_a_), 4.14 (t, *J* = 6.7 Hz, 12H, H_g_), 1.72 (p, *J* = 6.9 Hz, 12H, H_h_), 1.42–1.23 (m, 36H, H_i,j,k_), 0.85 (d, *J* = 7.1 Hz, 18H, H_l_). Diffusion coefficient (*d*_6_-DMSO, 298 K) D: 1.07 × 10^−10^ m^2^·s^−1^. ^13^C-NMR (125 MHz, CD_3_NO_2_, 298 K) δ: 155.3, 143.9, 137.8, 136.0, 123.6, 122.5, 122.5, 66.5, 56.8 (under residual solvent peak), 27.0, 24.2, 20.9, 18.1, 8.9. ^19^F-NMR (376 MHz, D_2_O, 298 K) δ: −78.9 (s). ATR-IR: ν (cm^−1^) 3112, 2929, 1589, 1570, 1446, 1370, 1249, 1110, 773, 636. HR-ESMS (DMSO/CH_3_CN) *m*/*z* = 564.2484 [M − 6OTf + CHOO + OH]^3+^ (calc. for C_82_H_110_Co_2_N_24_O_9_, 564.2512), 407.6917 [M − 6OTf]^4+^ (calc. for C_81_H_108_Co_2_N_24_O_6_, 407.6881). UV-Vis (DMF, ε [M^−1^·cm^−1^]): λ_max_ nm = 420 (8.62 × 10^4^).

**Synthesis of [Co_2_L3_3_](OTf)_6_:** [Co(OH_2_)_6_](BF_4_)_2_: 35 mg, 0.103 mmol, **L3**: 61 mg, 0.155 mmol, [Ce(NO_3_)_6_](NH_4_)_2_: 62 mg, 0.114 mmol, [N(C_4_H_9_)_4_](OTf): 607 mg, 1.551 mmol. Yellow-orange solid (67 mg, 31 μmol, 84%). ^1^H-NMR (500 MHz, CD_3_NO_2_, 298 K) δ: 9.44 (s, 1H, H_e_), 8.50–8.46 (m, 2H, H_c,d_), 7.86–7.68 (m, 2H, H_a,b_), 7.33 (s, 2H, H_g_), 5.74 & 5.54 (ABq, *J*_AB_ = 15.3 Hz, 2H, H_f_). Diffusion coefficient (*d*_6_-DMSO, 298 K) D: 1.12 × 10^−10^ m^2^·s^−1^. ^13^C-NMR (125 MHz, CD_3_NO_2_, 298 K) δ: 147.8, 144.8, 144.5, 140.1, 131.2, 125.4, 124.1, 124.0, 121.6, 52.3. ^19^F-NMR (376 MHz, D_2_O, 298 K) δ: −78.9 (s). ATR-IR: ν (cm^−1^) 3066, 1627, 1588, 1327, 1277, 1250, 1027, 756, 635. HR-ESMS (DMSO/CH_3_CN) *m*/*z* = 325.0928 [M − 6OTf]^4+^ (calc. for C_66_H_54_Co_2_N_24_, 325.0901), 454.1186 [M − 6OTf + CHOO + OH]^3+^ (calc. for C_67_H_56_Co_2_N_24_O_3_, 454.1205). UV-Vis (DMF, ε [M^−1^·cm^−1^]): λ_max_ nm = 449 (2.86 × 10^4^).

**Synthesis of [Co_2_L4_3_](OTf)_6_:** [Co(OH_2_)_6_](BF_4_)_2_: 20 mg, 0.057 mmol, **L4**: 51 mg, 0.086 mmol, [Ce(NO_3_)_6_](NH_4_)_2_: 35 mg, 0.063 mmol, [N(C_4_H_9_)_4_](OTf): 336 mg, 0.858 mmol. Yellow-orange solid (55 mg, 25 μmol, 69%). ^1^H-NMR (500 MHz, CD_3_NO_2_, 298 K) δ: 9.12 (s, 6H, H_e_), 8.36 (d, *J* = 8.9 Hz, 6H, H_d_), 7.98 (dd, *J* = 9.0, 2.5 Hz, 6H, H_c_), 7.43 (d, *J* = 2.4 Hz, 6H, H_a_), 7.29 (s, 12H, H_g_), 5.70 & 5.51 (ABq, *J*_AB_ = 15.2 Hz, 12H, H_f_), 4.10 (t, *J* = 6.6 Hz, 12H, H_h_), 1.71 (p, *J* = 6.8 Hz, 12H, H_i_), 1.40–1.22 (m, 24H, H_j,k,l_), 0.85 (t, *J* = 6.8 Hz, 18H, H_m_). Diffusion coefficient (*d*_6_-DMSO, 298 K) D: 0.96 × 10^−10^ m^2^·s^−1^. ^13^C-NMR (125 MHz, CD_3_NO_2_, 298 K) δ: 161.7, 151.2, 143.8, 143.2, 137.9, 130.8, 129.3, 128.7, 128.7, 73.1, 58.9, 33.7, 30.9, 27.6, 24.8, 15.6. ^19^F-NMR (376 MHz, D_2_O, 298 K) δ: −78.9 (s). ATR-IR: ν (cm^−1^) 2924, 1440, 1278, 1248, 1028, 635. HR-ESMS (DMSO/CH_3_CN) *m/z* = 654.6223 [M − 6OTf + CHOO + OH]^3+^ (calc. for C_103_H_128_Co_2_N_24_O_9_, 654.324), 475.4769 [M − 6OTf]^4+^ (calc. for C_102_H_126_Co_2_N_24_O_6_, 475.4741). UV-Vis (DMF, ε [M^−1^·cm^−1^]): λ_max_ nm = 400 (1.25 × 10^5^). 

**Synthesis of [Fe_2_L1_3_](BF_4_)_4_:** A solution of **L1** (4 mg, 13 µmol) and [Fe(H_2_O)_6_](BF_4_)_2_ (3 mg, 9 µmol) in acetonitrile (0.5 mL) was filtered through cotton wool, before vapor diffusion with diethyl ether yielded a red crystalline solid (3 mg, 2 µmol, 78%). ^1^H-NMR (500 MHz, CD_3_CN, 298 K) δ: 8.99 (6H, s, H_e_), 8.23 (6H, s, H_b_), 8.10 (6H, s, H_c_), 8.03 (6H, s, H_a_), 7.46 (6H, s, H_b_), 6.77 & 6.46 (6H, ABq, *J*_AB_ = 17.6 Hz, H_f_). ^13^C-NMR (125 MHz, CD_3_CN, 298 K) δ: 155.9, 153.0, 150.3, 140.1, 126.8, 125.6, 123.5, 62.4. Mass spectral analysis was not successful for this compound. Anal. calcd. for C_45_H_36_B_4_F_16_Fe_2_N_24_∙4H_2_O: C, 37.43; H, 3.07; N, 23.28%. Found: C, 37.66; H, 2.67; N, 22.94%.

## Supplementary Materials

Supplementary materials can be accessed at: http://www.mdpi.com/1420-3049/21/11/1548/s1. ^1^H-, ^13^C-NMR, UV-Vis and HR-ESMS spectral data, biological procedures, and crystallographic data in CIF format.
