# Supplementary material for: Oxidatively Locked [Co2L3]6+ Cylinders Derived from Bis(bidentate) 2-Pyridyl-1,2,3-triazole “Click” Ligands: Synthesis, Stability, and Antimicrobial Studies
Source: Molecules. 2016 Nov 16;21(11):1548. doi: 10.3390/molecules21111548 (PMC6273053; doi:10.3390/molecules21111548)
Supplement: Supplementary file 1 [file molecules-21-01548-s001.pdf]

Supplementary Materials: Oxidatively Locked $[Co_2L_3]^{6+}$ Cylinders derived from Bis(bidentate) 2-Pyridyl-1,2,3-triazole "Click" Ligands: Synthesis, Stability and Antimicrobial Studies

Roan A. S. Vasdev, Dan Preston, Synøve Ø. Scottwell, Heather J. L. Brooks, James D. Crowley and Michael P. Schramm

1. Experimental

1.1. General

Unless otherwise stated, all reagents were purchased from commercial sources and used without further purification. The ligands **L1**, **L2**, and **L3** were synthesised using our previously reported procedures [1,2]. Solvents were laboratory reagent grade. Petroleum ether refers to the fraction of petrol boiling in the range 40–60 °C. Substances (and abbreviations) used in this study include isopropyl alcohol (IPA), methanol (CH_3OH), dichloromethane (CH_2Cl_2), ethylenediaminetetraacetate (EDTA), ethynyltrimethylsilane (TMS-acetylene), tetrahydrofuran (THF), dimethyl sulfoxide (DMSO), dimethylformamide (DMF). ^{1}H- and ^{13}C-NMR spectra were recorded on either a 400 MHz Varian 400-MR or a Varian 500 MHz AR spectrometer (Varian, Santa Clara, CA, USA). Chemical shifts are reported in parts per million and referenced to residual solvent peaks ($CDCl_3$: ^{1}H δ 7.26 ppm, ^{13}C δ 77.16 ppm; CD_3CN: ^{1}H δ 1.94, ^{13}C δ 1.32, 118.26 ppm, *d*$_6$-DMSO: ^{1}H δ 2.50 ppm; ^{13}C δ 39.52 ppm; CD_3NO_2: ^{1}H δ 4.33, ^{13}C δ 57.3 ppm). Coupling constants (*J*) are reported in Hertz (Hz). Standard abbreviations indicating multiplicity were used as follows: m = multiplet, t = triplet, q = quartet, quin = quintet, dt = double triplet, d = doublet, dd = double doublet, s = singlet, and br = broad. IR spectra were recorded on a Bruker ALPHA FT-IR spectrometer with an attached ALPHA-P measurement module. A CEM S-class microwave reactor was used to carry out microwave enhanced reactions. Microanalyses were performed at the Campbell Microanalytical Laboratory at the University of Otago. Electrospray mass spectra (ESMS) were collected on a Bruker micrOTOF-Q spectrometer (Bruker, Billerica, MA, USA). UV-Vis spectra were recorded on a Perkin Elmer Lambda 950 UV/Vis/NIR spectrometer (Waltham, MA, USA).

1.2. **L4**

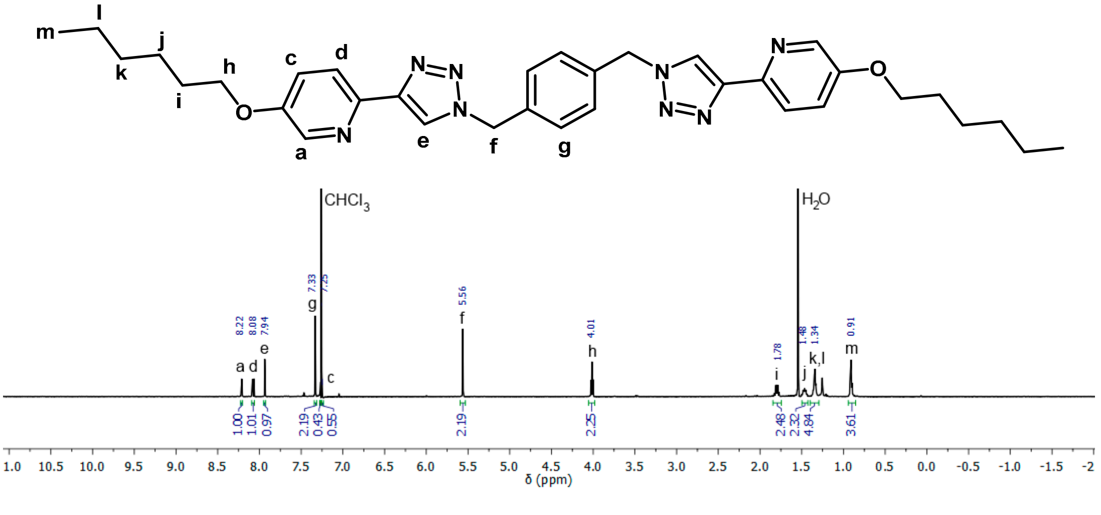

Figure S Error! No text of specified style in document.**.** ^{1}H-NMR (400 MHz, $CDCl_3$, 298 K) of **L4**.

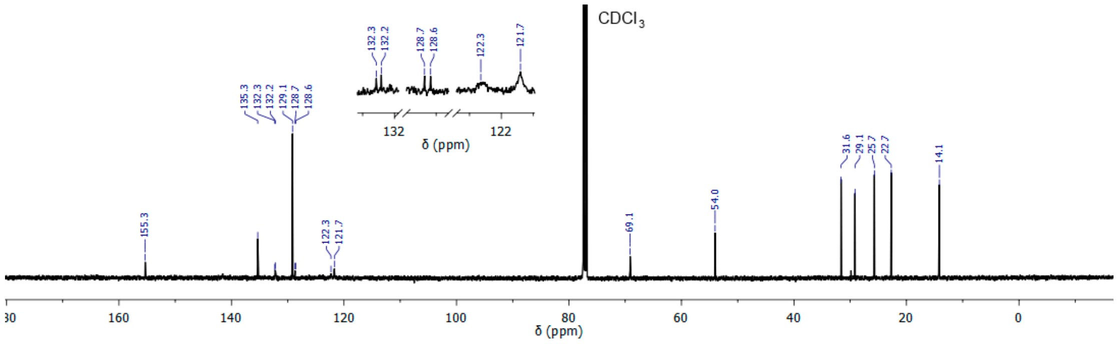

Figure S1. ^{13}C-NMR (125 MHz, $CDCl_3$, 298 K) of **L4**.

1.3. [Co$_2$**L2**$_3$](BF$_4$)$_4$

In the ^{1}H-NMR spectrum of the isolated intermediate, [Co$_2$**L2**$_3$](BF$_4$)$_4$, the chemical shifts of the proton signals are far greater than those observed for [Co$_2$**L2**$_3$](OTf)$_6$, and there is no splitting of the peaks. This indicates that the paramagnetic Co(II) cylinder has been formed [3]. The insert (−3–10 ppm) in the figure below shows the absence of "free" ligand.

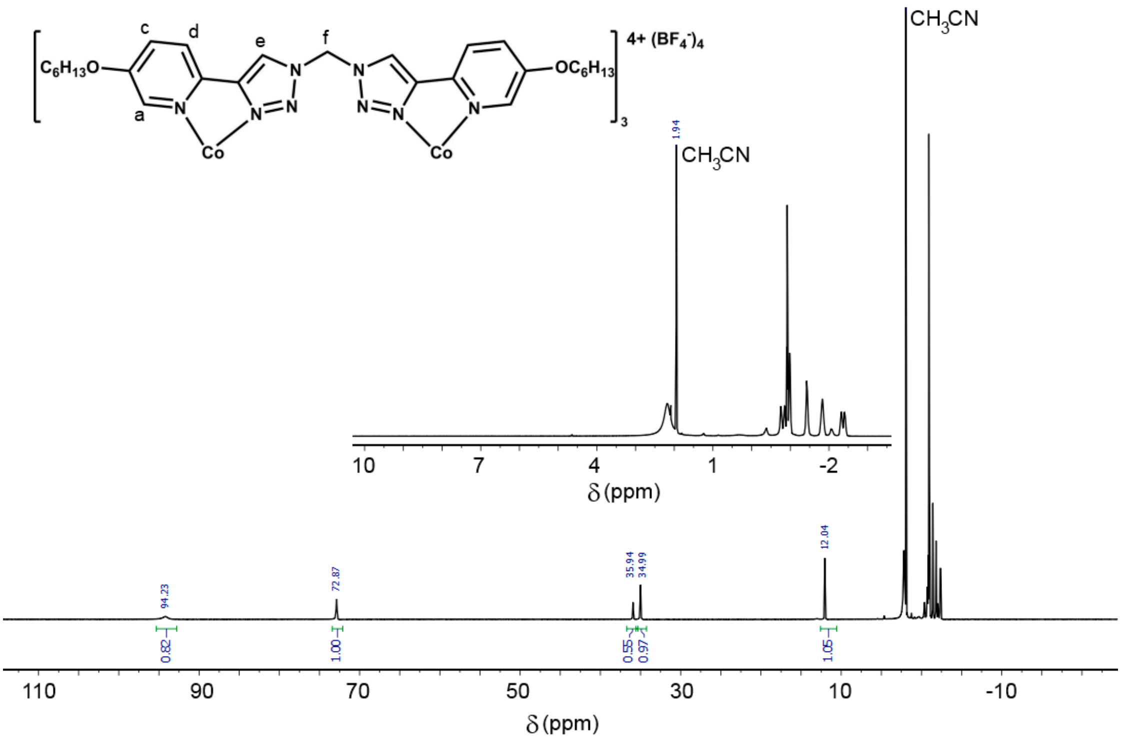

Figure S2. ^{1}H-NMR (400 MHz, CD_3CN, 298 K) of [Co$_2$**L2**$_3$](BF$_4$)$_4$.

1.4. [Co2**L1**3](PF6)6

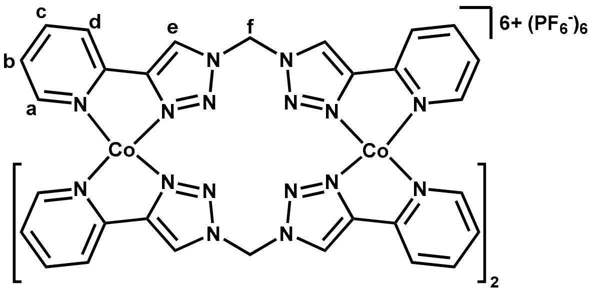

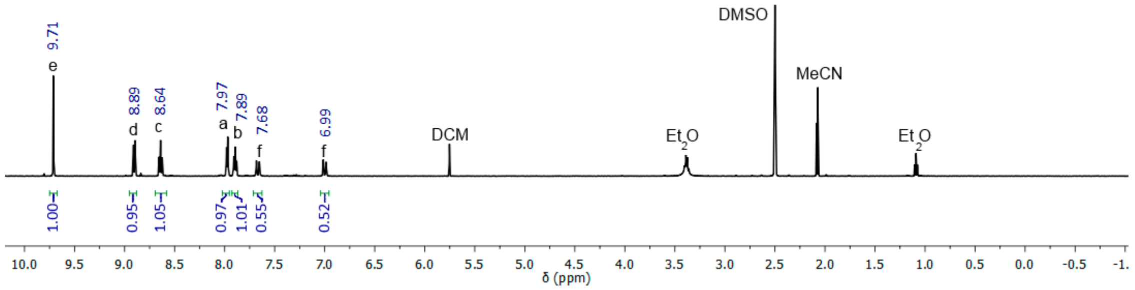

Figure S4. ^{1}H-NMR (400 MHz, CD_3CN, 298 K) of [Co_2**L1**$_3$](PF_6)$_6$.

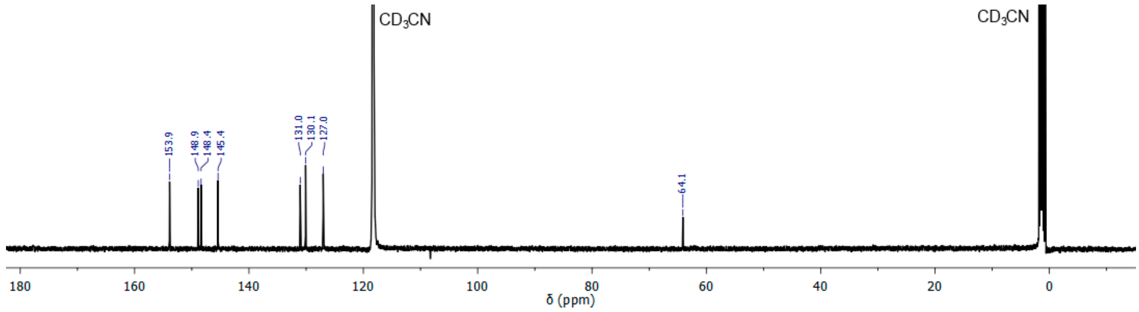

Figure S5. ^{13}C-NMR (125 MHz, CD_3CN, 298 K) of [Co_2**L1**$_3$](PF_6)$_6$.

1.5. [Co2**L1**3](OTf)6

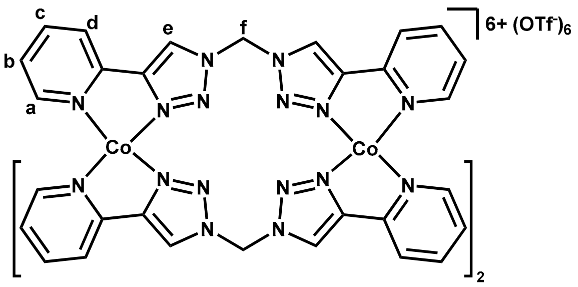

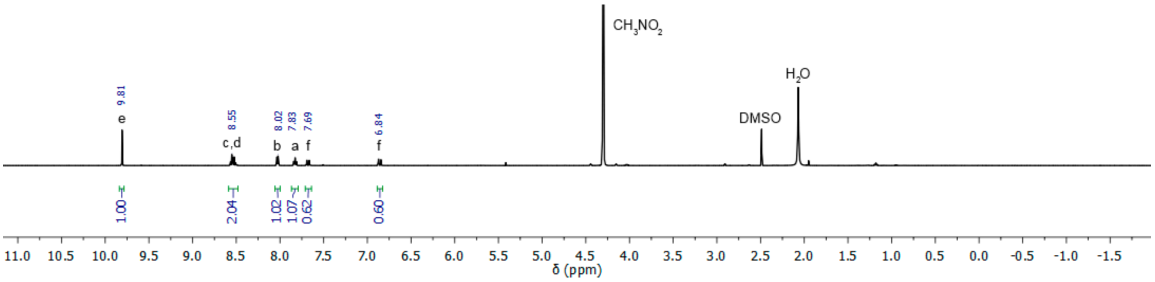

Figure S6. ^{1}H-NMR (500 MHz, CD_3NO_2, 298 K) of [Co_2**L1**$_3$](OTf)$_6$.

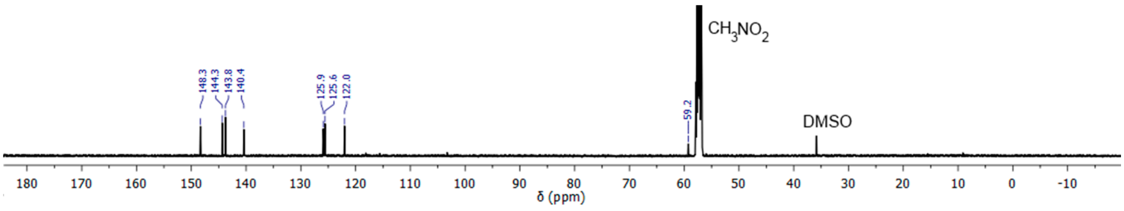

Figure S7. ^{13}C-NMR (125 MHz, CD_3NO_2, 298 K) of [Co_2**L1**$_3$]$(OTf)_6$.

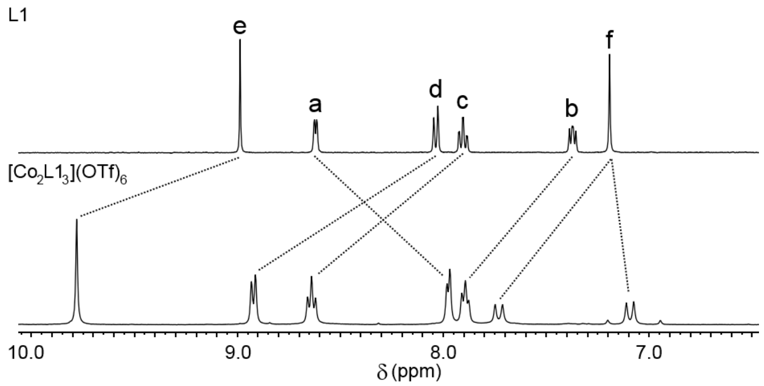

Figure S8. ^1H-NMR (400 MHz, d_6-DMSO, 298 K) stacked spectra of **L1** and [Co_2**L1**$_3$]$(OTf)_6$.

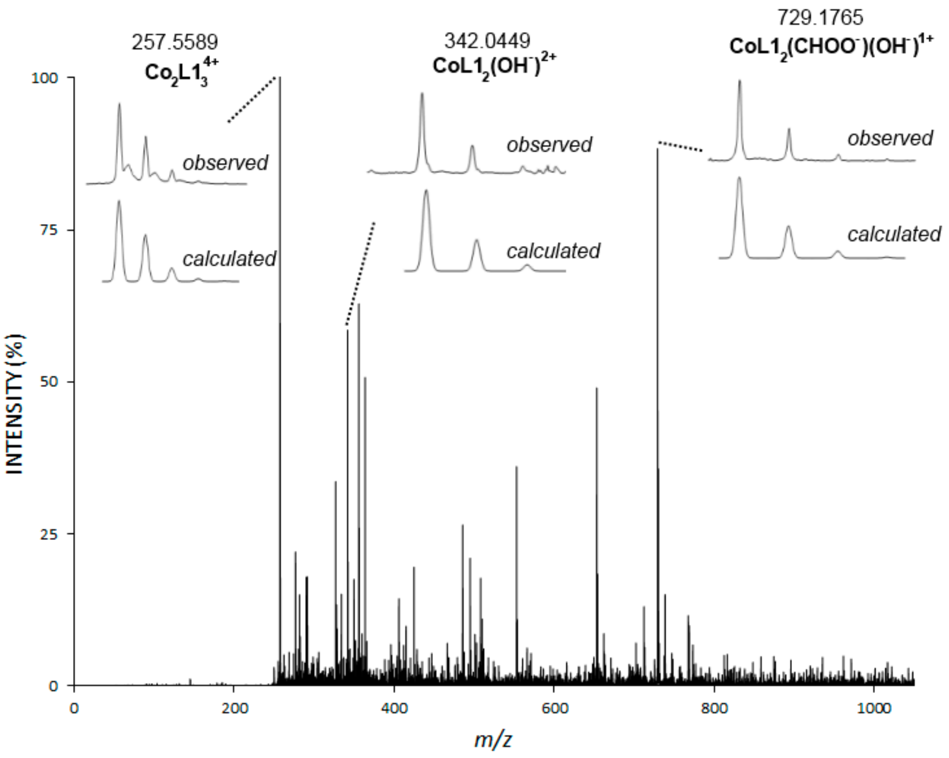

Figure S9. HR ESI-MS ($DMSO/CH_3CN$) of [Co_2**L1**$_3$]$(OTf)_6$.

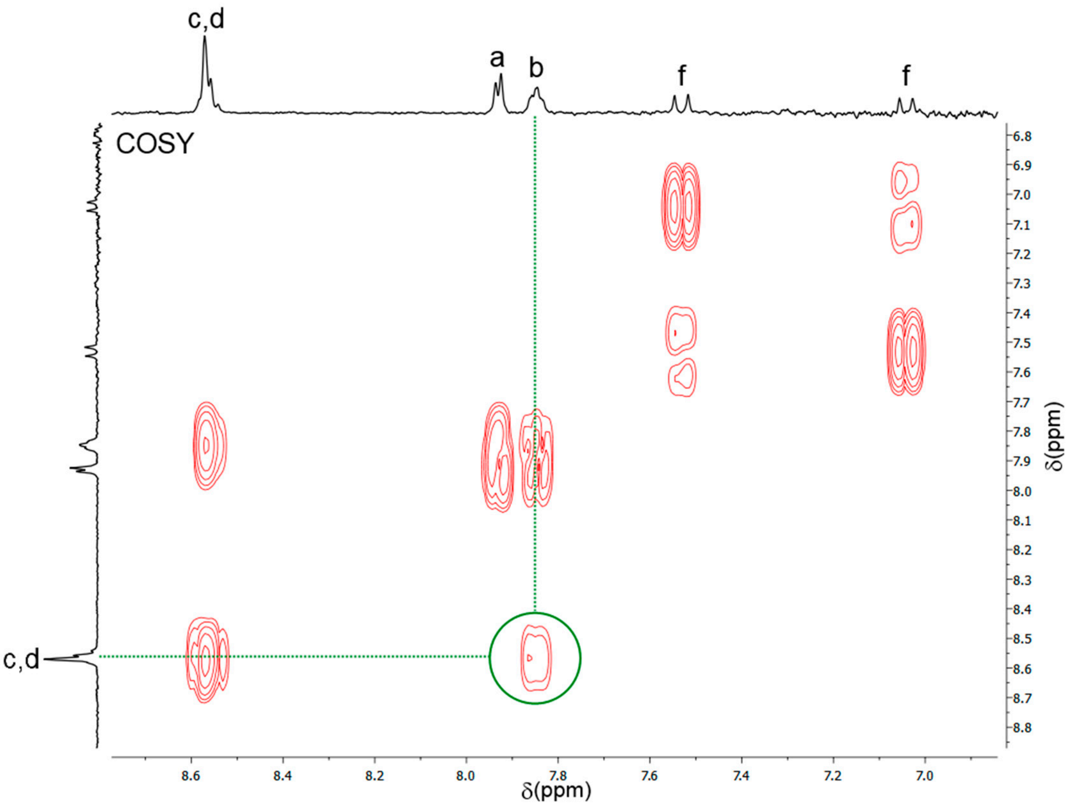

Figure S10. COSY ^{1}H-NMR (500 MHz, D_2O, 298 K) of [Co_2**L1**$_3$]$(OTf)_6$ showing coupling between H_b and H_c.

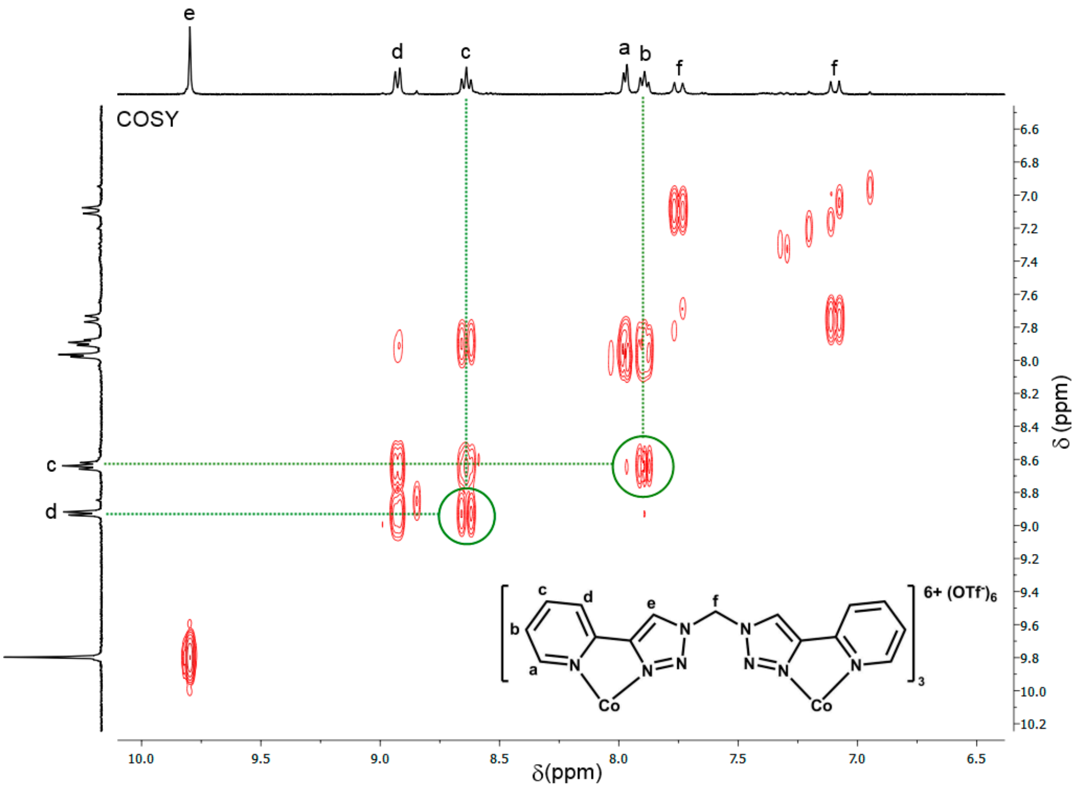

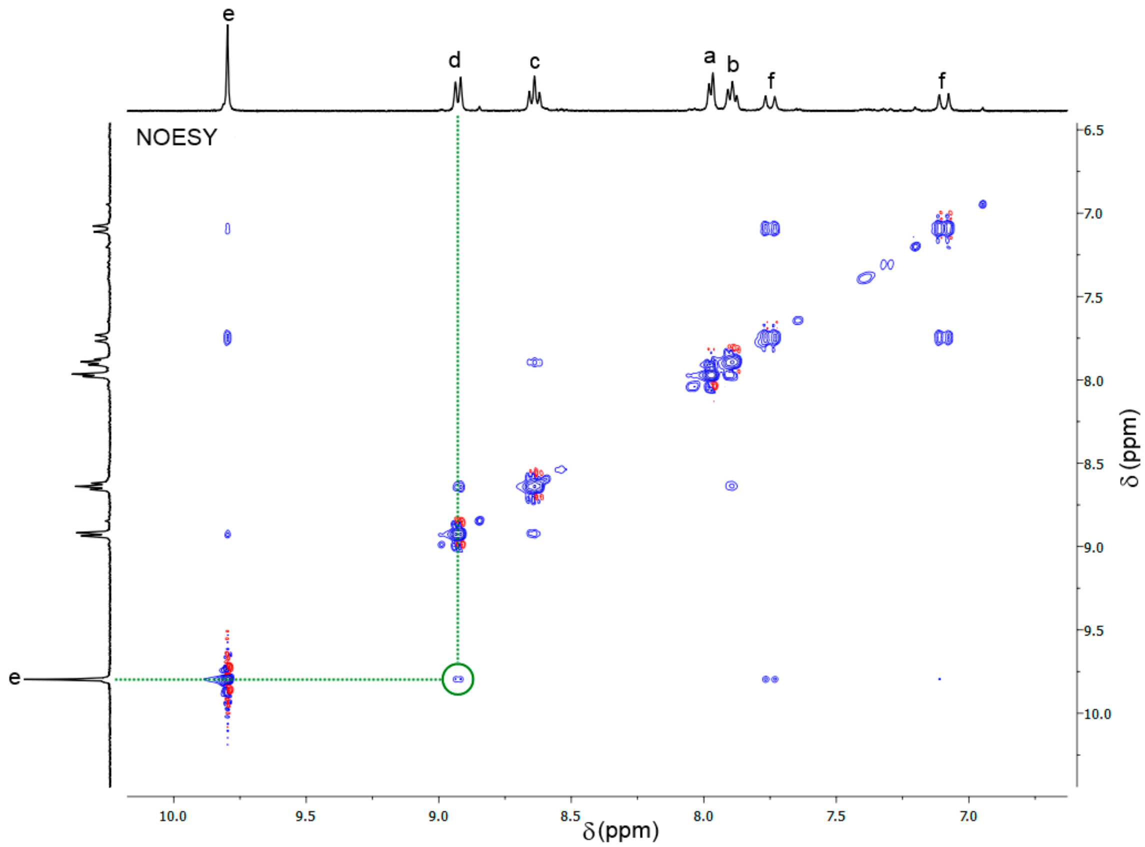

Figure S11. COSY and NOESY ^{1}H-NMR (400 MHz, d_6-DMSO, 298 K) of [Co_2**L1**$_3$]$(OTf)_6$ showing coupling between H_c and H_d, H_b and H_c (COSY), and H_d and H_e (NOESY).

1.6. [Co_2**L2**$_3$]$(OTf)_6$

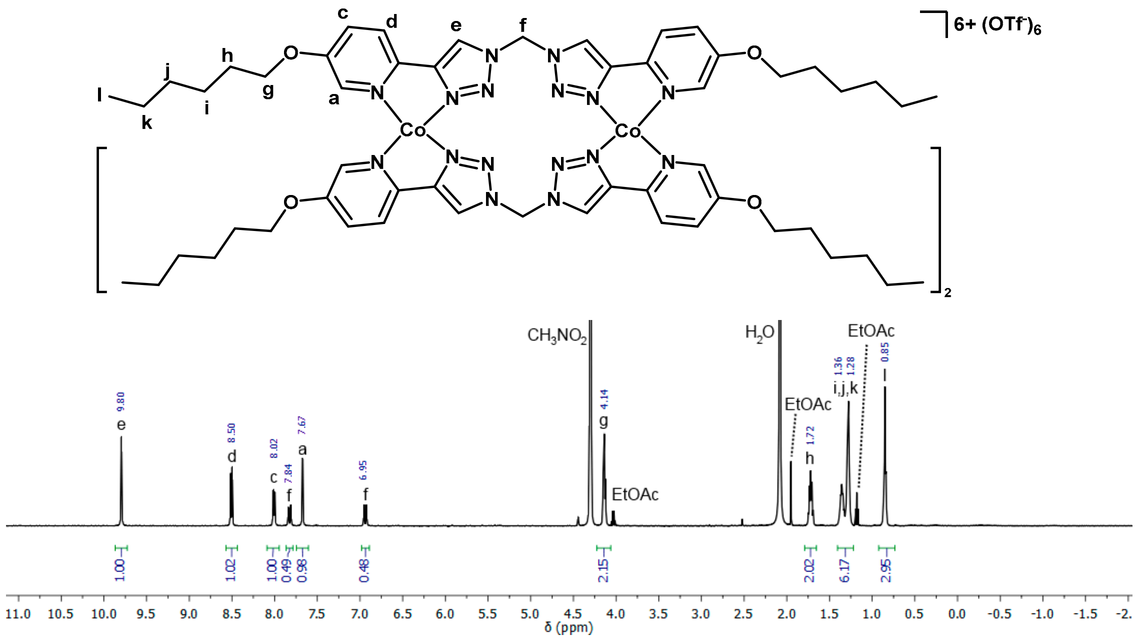

Figure S12. ^{1}H-NMR (500 MHz, CD_3NO_2, 298 K) of [Co_2**L2**$_3$]$(OTf)_6$.

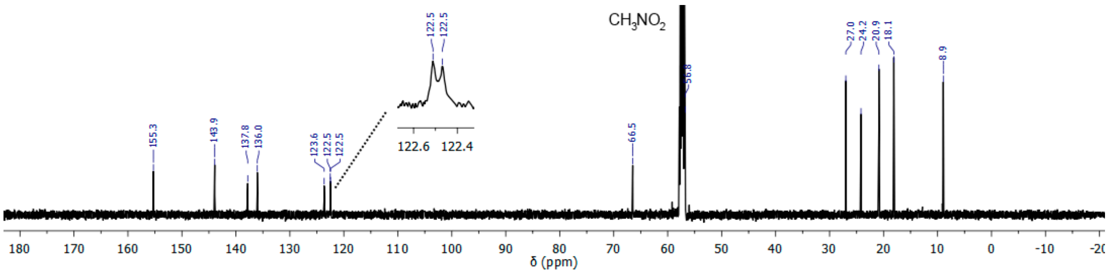

Figure S13. ^{13}C-NMR (125 MHz, CD_3NO_2, 298 K) of [Co_2**L2**$_3$]$(OTf)_6$.

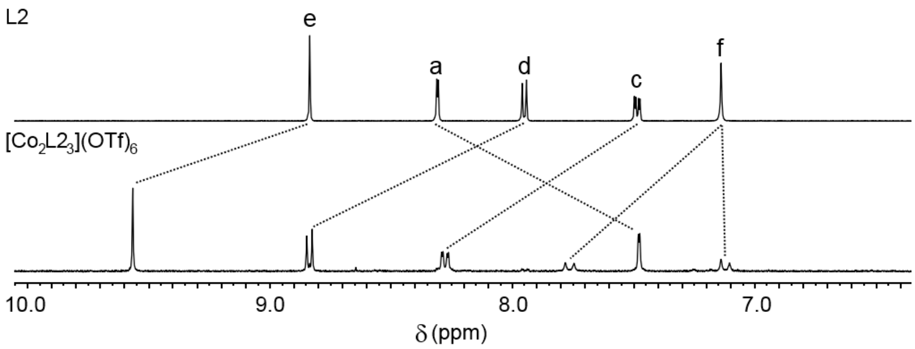

Figure S14. ^{1}H-NMR (400 MHz, *d*$_6$-DMSO, 298 K) stacked spectra of **L2** and [Co_2**L2**$_3$]$(OTf)_6$.

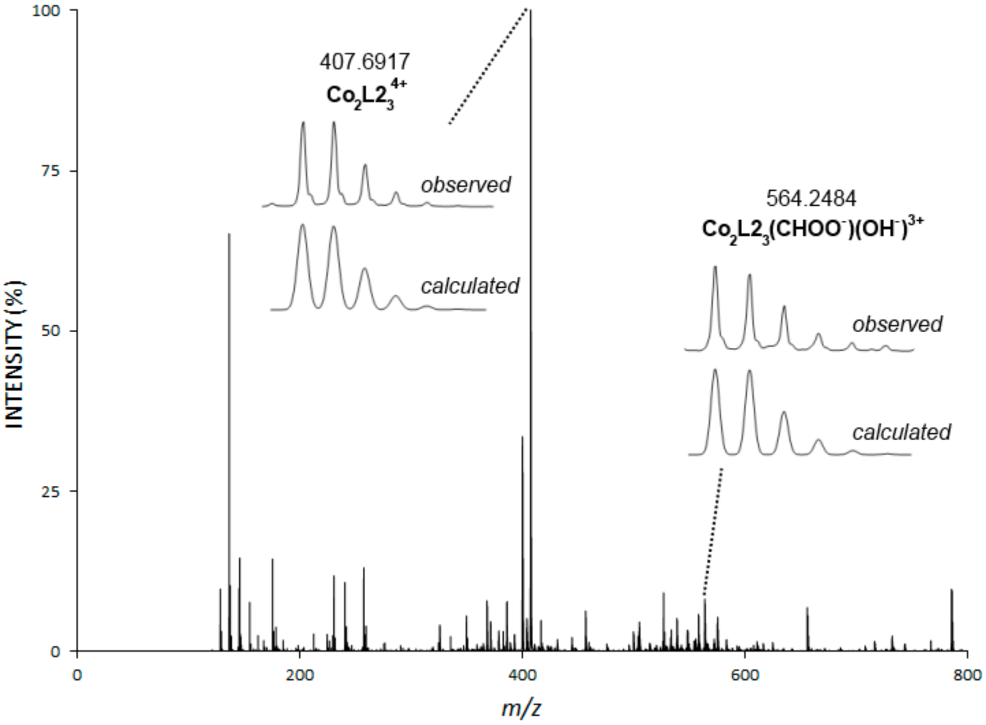

Figure S15. HR ESI-MS (DMSO/CH_3CN) of [Co_2**L2**$_3$]$(OTf)_6$.

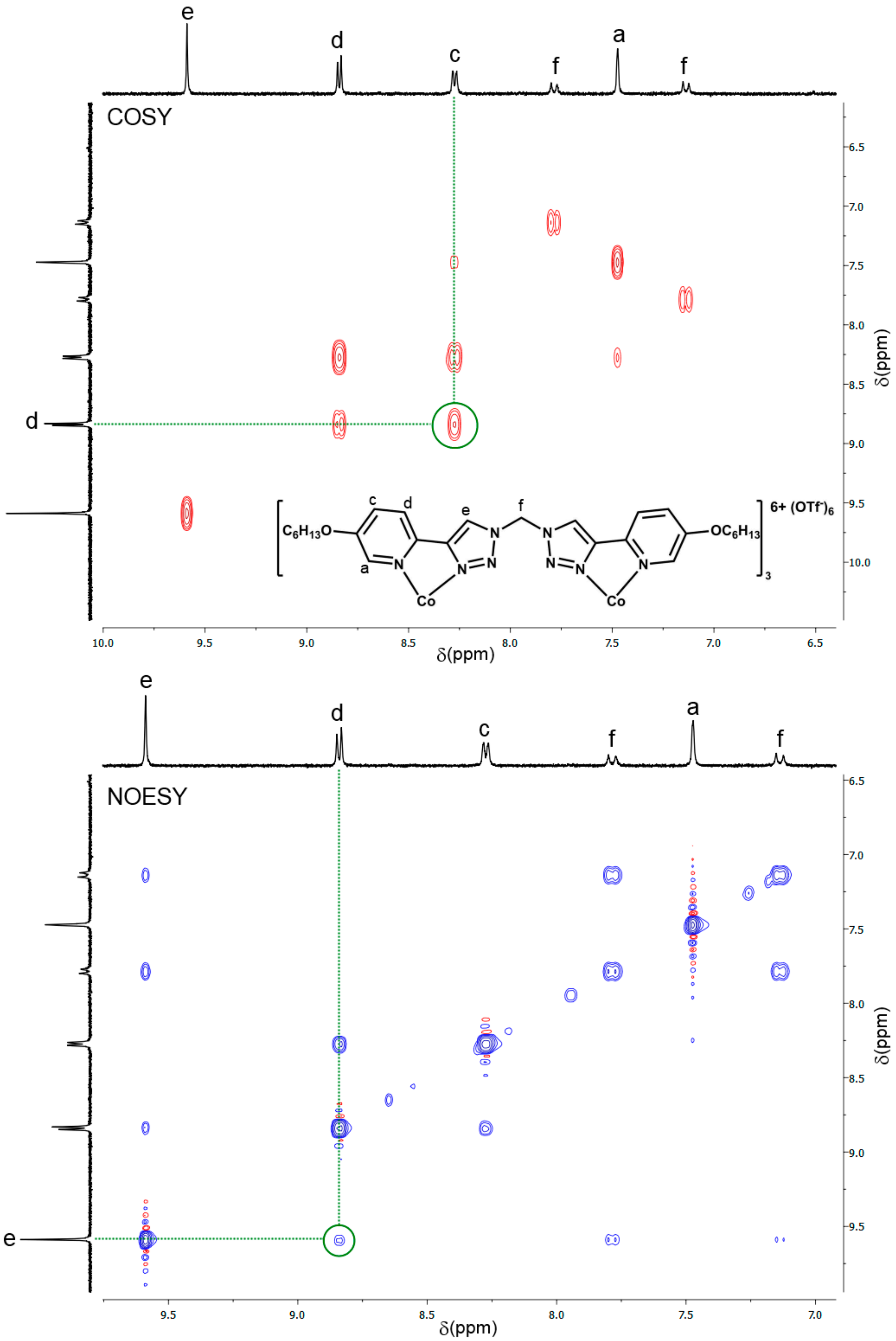

Figure S16. COSY and NOESY ^{1}H-NMR (500 MHz, d_6-DMSO, 298 K) of $[Co_2\mathbf{L2}_3](OTf)_6$ showing coupling between H_c and H_d (COSY) and H_d and H_e (NOESY).

1.7. [Co$_2$**L3**$_3$](OTf)$_6$

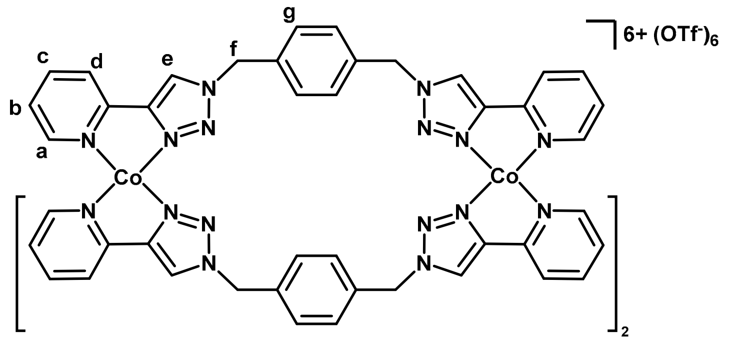

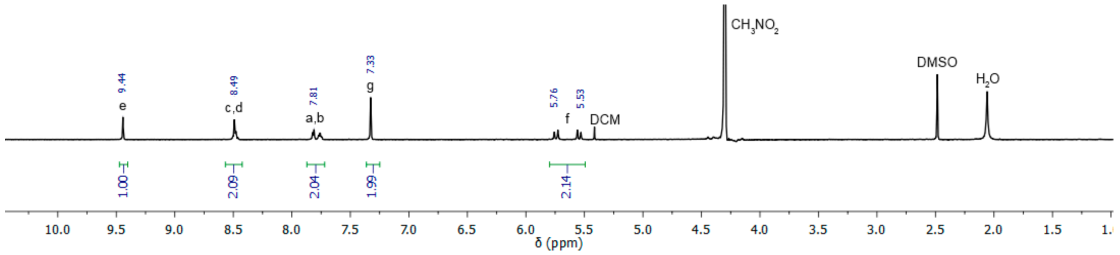

Figure S17. ^{1}H-NMR (500 MHz, CD_3NO_2, 298 K) of [Co$_2$**L3**$_3$](OTf)$_6$.

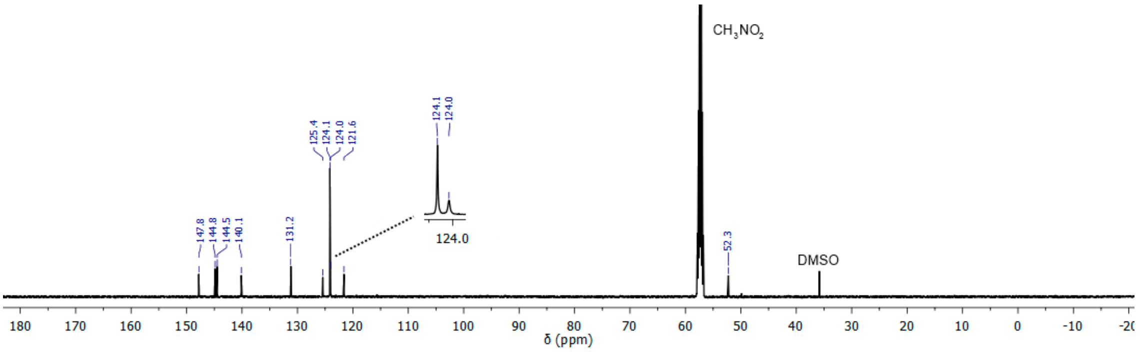

Figure S18. ^{13}C-NMR (125 MHz, CD_3NO_2, 298 K) of [Co$_2$**L3**$_3$](OTf)$_6$.

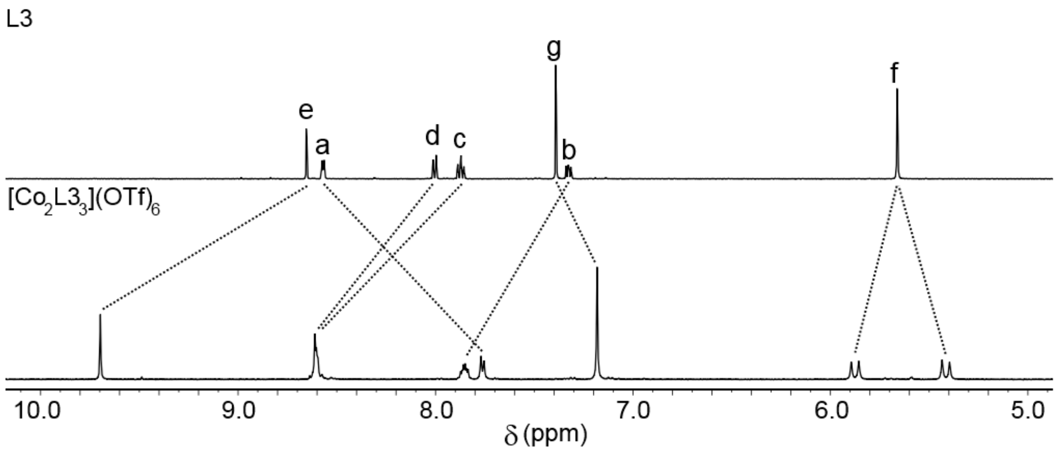

Figure S19. ^{1}H-NMR (400 MHz, *d*$_6$-DMSO, 298 K) stacked spectra of **L3** and [Co$_2$**L3**$_3$](OTf)$_6$.

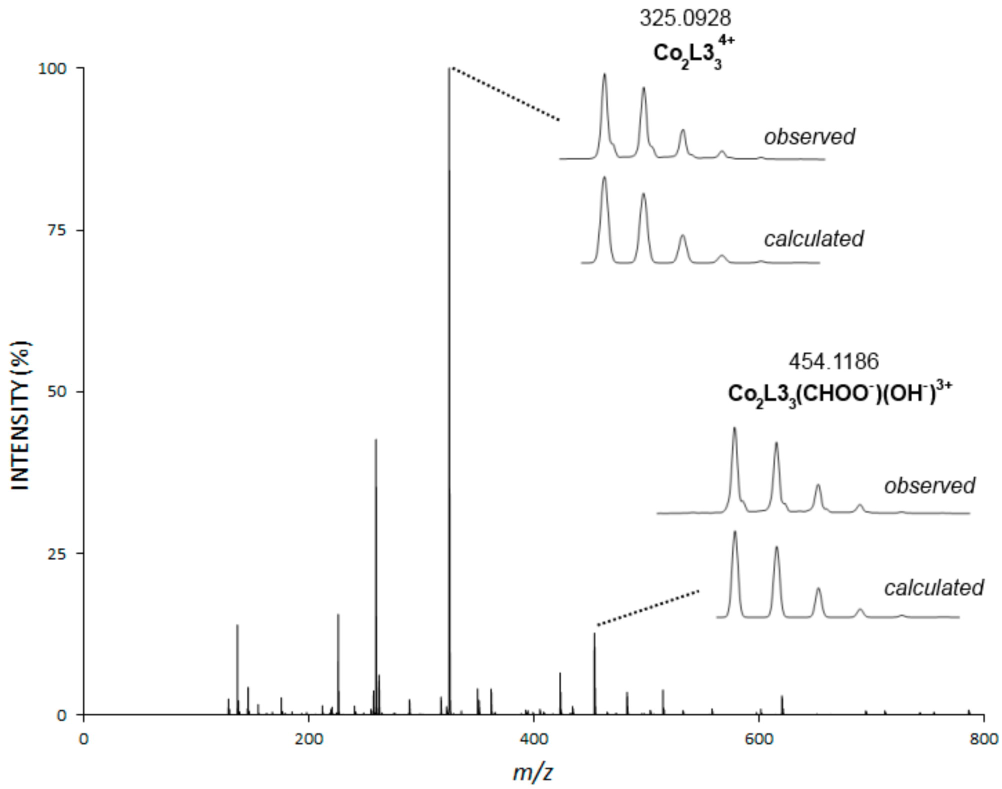

Figure S20. HR ESI-MS (DMSO/CH$_3$CN) of [Co$_2$**L3**$_3$](OTf)$_6$.

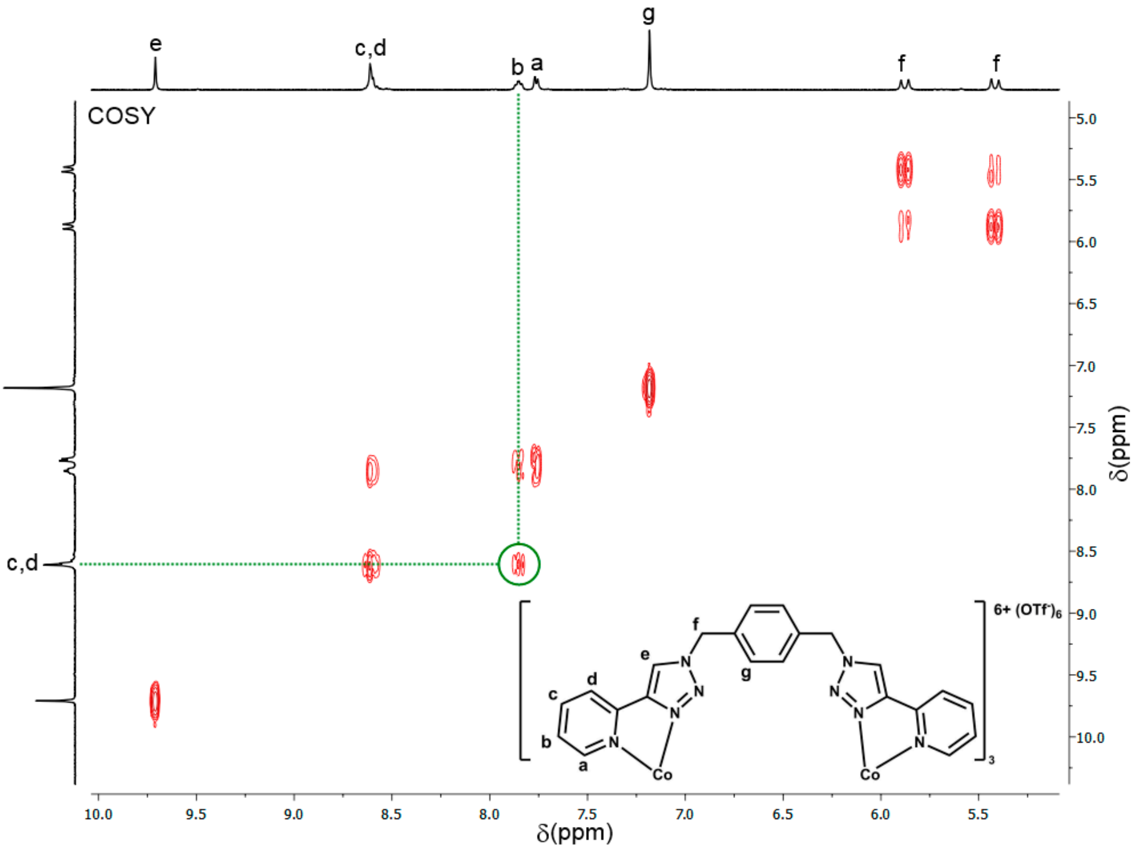

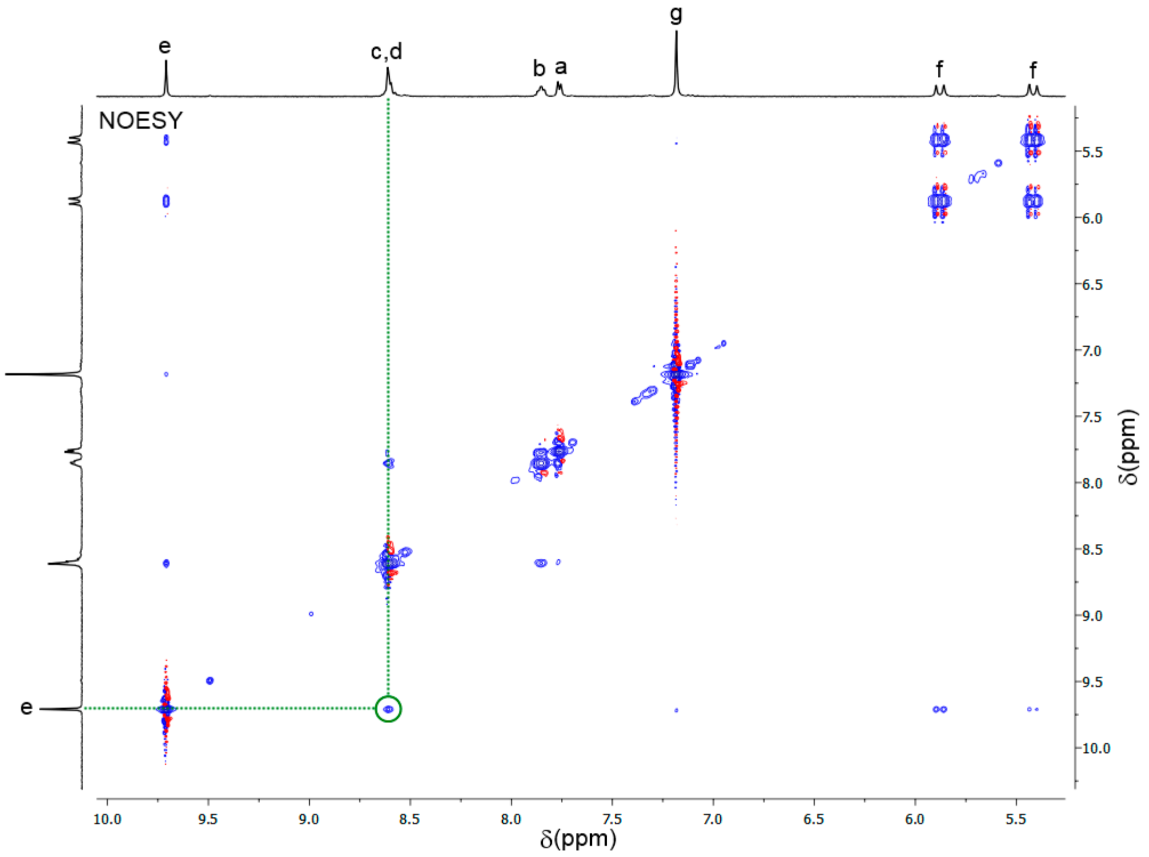

Figure S21. COSY and NOESY ^{1}H-NMR (400 MHz, d_6-DMSO, 298 K) of $[Co_2\mathbf{L3}_3](OTf)_6$ showing coupling between H_b and H_c (COSY) and H_d and H_e (NOESY).

1.8. $[Co_2\mathbf{L4}_3](OTf)_6$

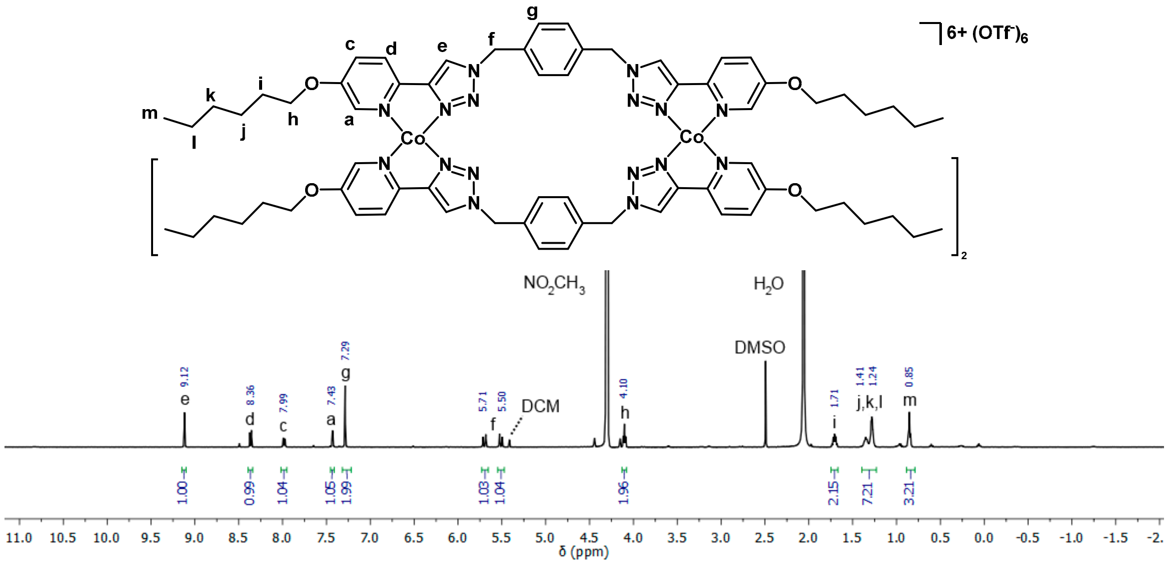

Figure S22. ^{1}H-NMR (500 MHz, CD_3NO_2, 298 K) of $[Co_2\mathbf{L4}_3](OTf)_6$.

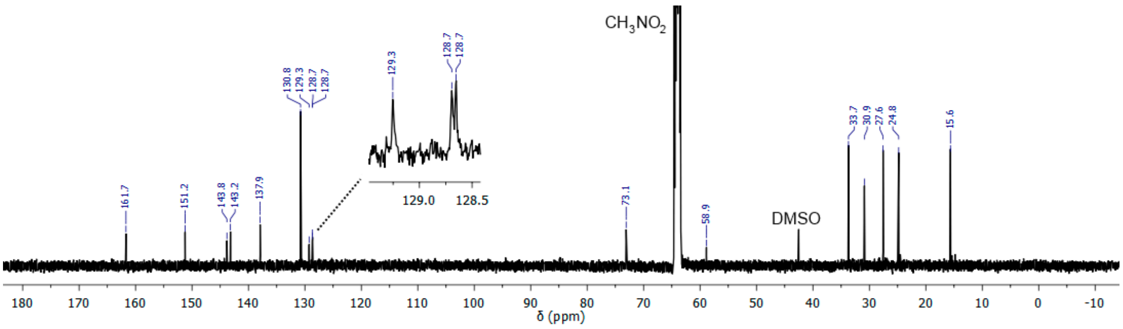

Figure S23. ^{13}C-NMR (125 MHz, CD_3NO_2, 298 K) of $[Co_2\mathbf{L4}_3](OTf)_6$.

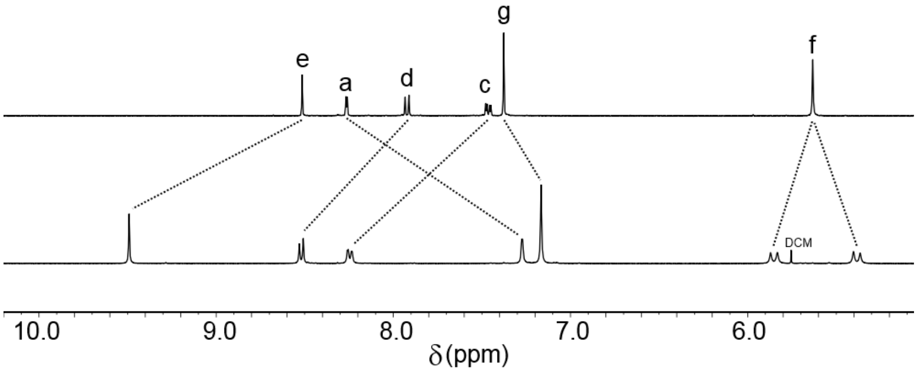

Figure S24. ^{1}H-NMR (400 MHz, d_6-DMSO, 298 K) stacked spectra of **L4** and $[Co_2\mathbf{L4}_3](OTf)_6$.

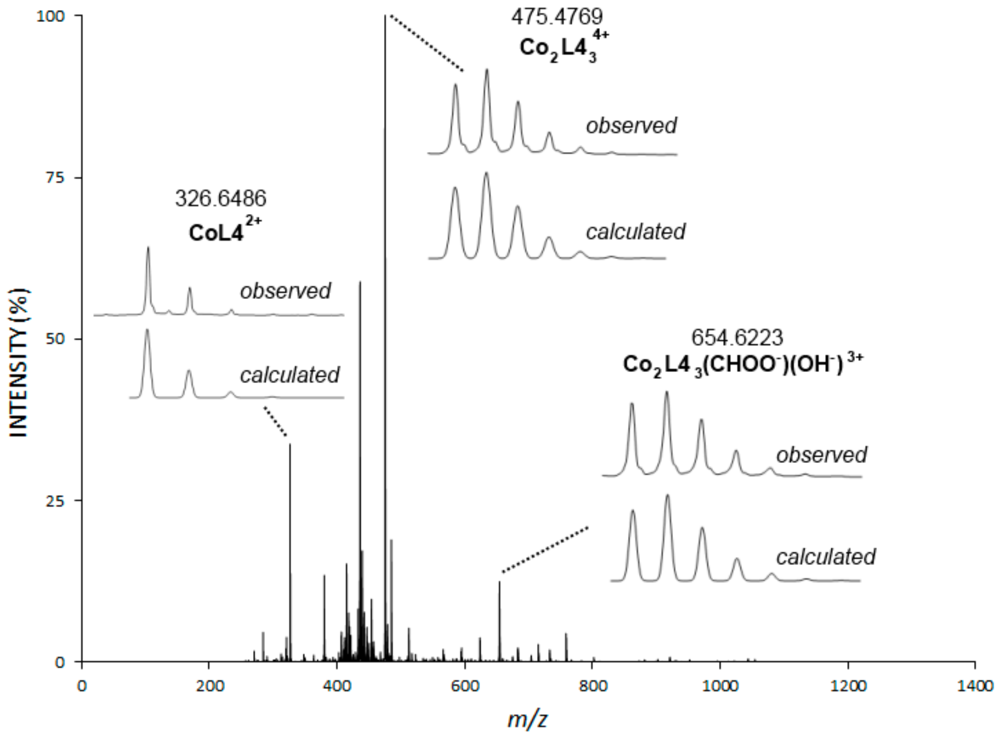

Figure S25. HR ESI-MS (DMSO/CH_3CN) of $[Co_2\mathbf{L4}_3](OTf)_6$.

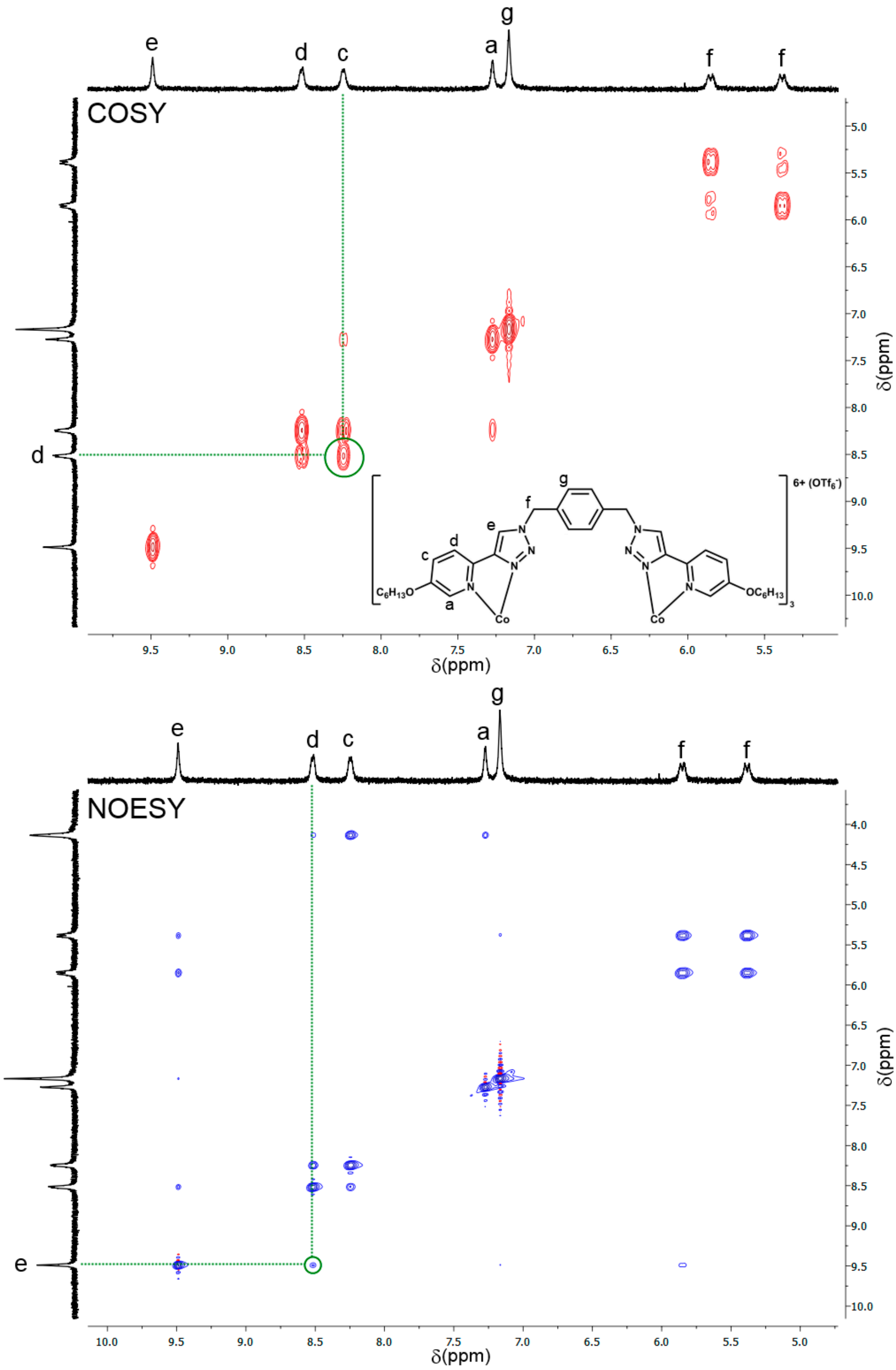

Figure S26. COSY and NOESY ^{1}H-NMR (500 MHz, d_6-DMSO, 298 K) of [Co_2**L4**$_3$]$(OTf)_6$ showing coupling between H_c and H_d (COSY) and H_d and H_e (NOESY).

1.9. ¹H Diffusion-Ordered Spectroscopy (DOSY)

Diffusion coefficients (*D*, $\times 10^{-10}$ $m^2 \cdot s^{-1}$) measured in d_6-DMSO at 500 MHz and 298 K. The cobalt cylinders and their ligands were combined and log(*D*) was plotted against log(M_W).

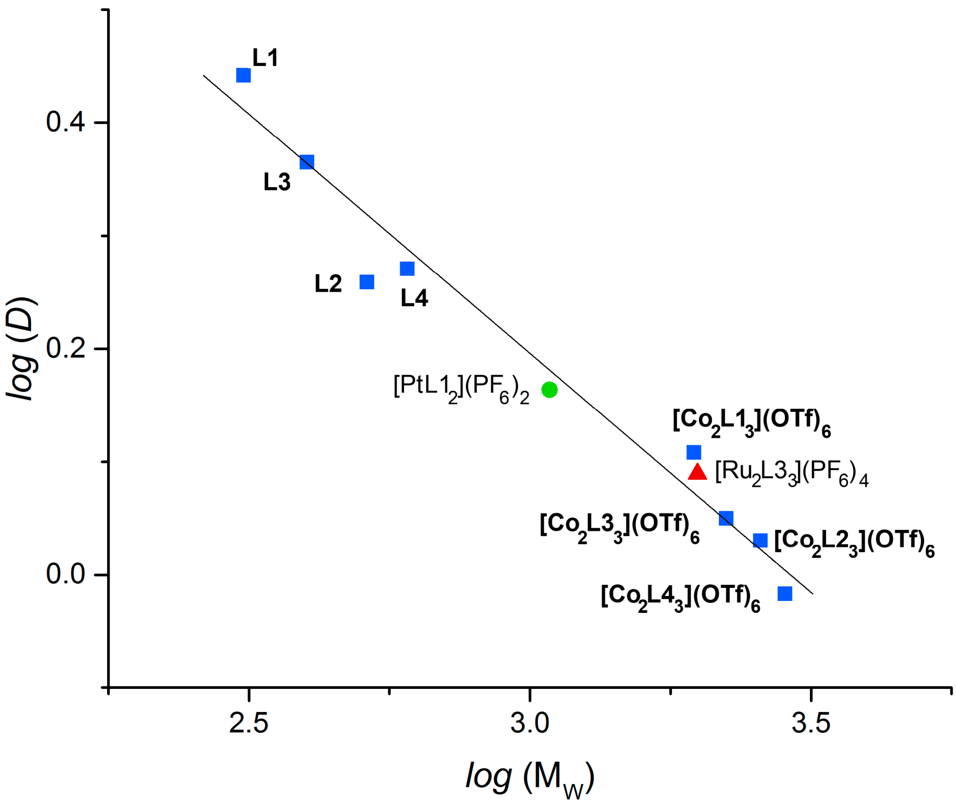

Figure S27. Plot of log(*D*) against log (MW) for Co(III) complexes, a Pt(II) complex, [1] a Ru(II) complex, [4] and ligands (500 MHz, d_6-DMSO, 298 K), diffusion coefficients: $\times 10^{-10}$ $m^2 \cdot s^{-1}$ and molecular weights: $g \cdot mol^{-1}$. The linear fit to the data gives the equation: log(*D*) = 3.45 − 2.36log(M_W) with an R^2 of 0.962.

Table S1. ¹H DOSY NMR data—derived diffusion coefficients (*D*) for compounds (500 MHz, d_6-DMSO, 298 K).

Compound	Diffusion Coefficient (*D*) × 10^{-10} $m^2 \cdot s^{-1}$	Molecular Weight (M_W) $g \cdot mol^{-1}$
**L1**	2.76	304.32
**L2**	1.81	504.64
**L3**	2.31	394.44
**L4**	2.17	594.79
$[Co_2$**L1**$_3](OTf)_6$	1.28	1925.2
$[Co_2$**L2**$_3](OTf)_6$	1.07	2526.16
$[Co_2$**L3**$_3](OTf)_6$	1.12	2195.57
$[Co_2$**L4**$_3](OTf)_6$	0.96	2796.54
$[Pt$**L1**$_2](PF_6)_2$	1.46	1093.14
$[Ru_2$**L3**$_3](PF_6)_4$	1.23	1965.32

1.10. [Fe$_2$**L1**$_3$](BF$_4$)$_4$

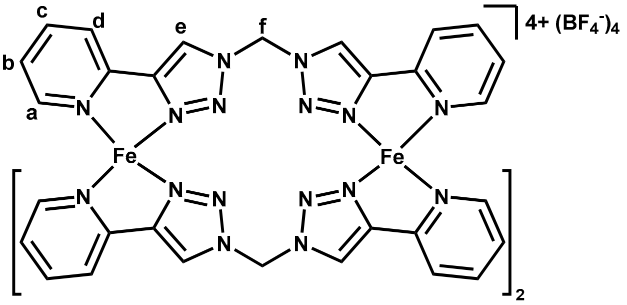

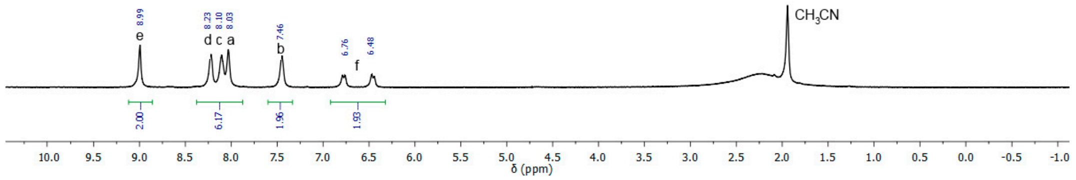

Figure S28. ^{1}H-NMR (500 MHz, CD$_3$CN, 298 K) of [Fe$_2$**L1**$_3$](BF$_4$)$_4$.

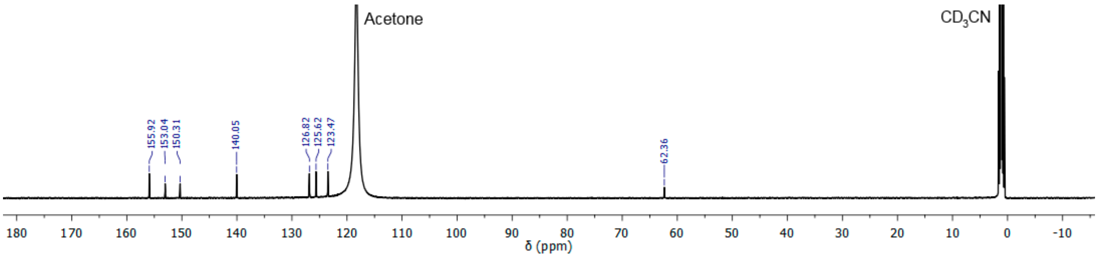

Figure S29. ^{13}C-NMR (125 MHz, CD$_3$CN, 298 K) of [Fe$_2$**L1**$_3$](BF$_4$)$_4$.

1.11. ^{1}H-NMR Data

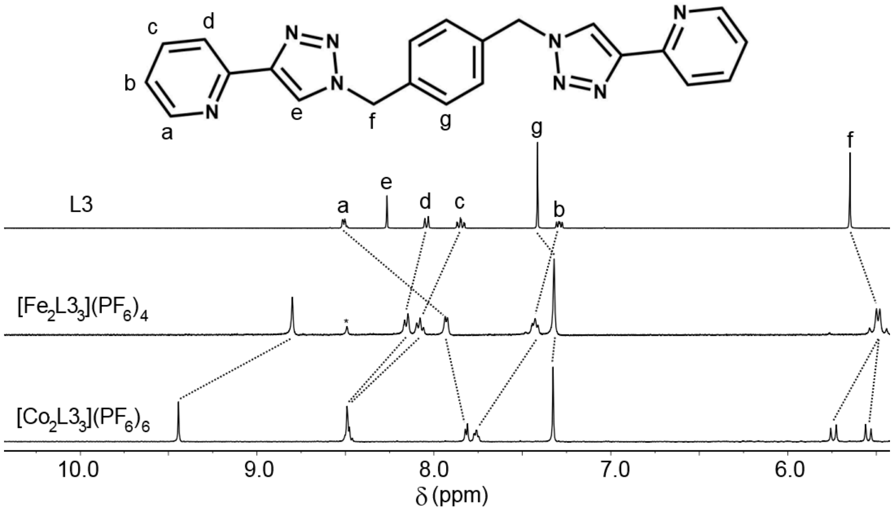

Figure S30. ^{1}H-NMR (400 MHz, CH$_3$NO$_2$) spectra of **L3**, [Fe$_2$**L3**$_3$](PF$_6$)$_4$ and [Co$_2$**L3**$_3$](PF$_6$)$_6$. *CH$_3$NO$_2$ impurity.

1.12. UV-Vis Spectra

Spectra were obtained in dimethylformamide (DMF) at 298 K at a concentration of ~0.1 mmolar. These values are consistent with the literature [5].

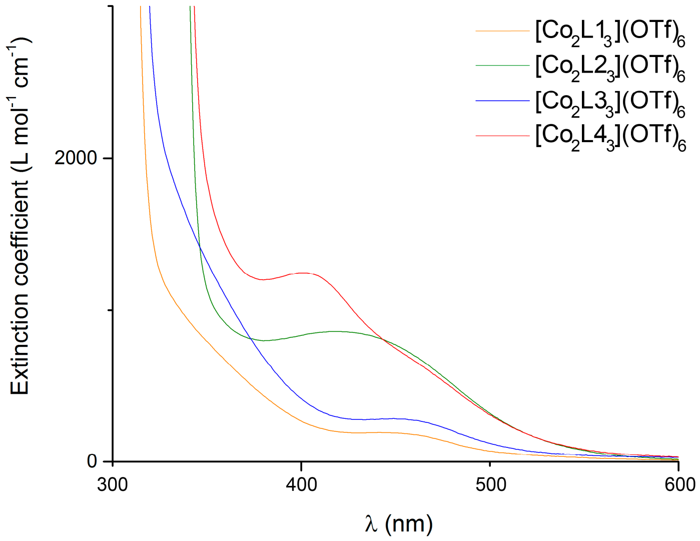

Figure S31. UV-Vis spectra of $[Co_2$**L1**$_3](OTf)_6$, $[Co_2$**L2**$_3](OTf)_6$, $[Co_2$**L3**$_3](OTf)_6$, $[Co_2$**L4**$_3](OTf)_6$.

2. X-ray Data

2.1. $[Fe_2$**L1**$_3](BF_4)_4$·4.4CH_3CN

Vapor diffusion of diethyl ether into a solution of $[Fe_2$**L1**$_3](BF_4)_4$ in acetonitrile gave red crystals of two morphologies: hexagons which during screening indicated a hexagonal space group but did not solve, and red blocks of $[Fe_2$**L1**$_3](BF_4)_4$·4.4CH_3CN. X-ray data were collected at 100 K on an Agilent Technologies Supernova system using Cu Kα radiation with exposures over 1.0°, and data were treated using CrysAlisPro [6] software. The structure was solved using Sir-97 [7] and weighted full-matrix refinement on F^2 was carried out using SHELXL-97 [8] running within the WinGX package [9]. All non-hydrogen atoms were refined anisotropically. Hydrogen atoms attached to carbons were placed in calculated positions and refined using a riding model. The structure was solved in the primitive triclinic space group $P\bar{1}$ and refined to an R_1 value of 9.7%. The asymmetric unit contains the diiron(II) mesocate, 4 tetrafluoroborate anions, and 4.4CH_3CN solvent molecules (**Error! Reference source not found.**S32).

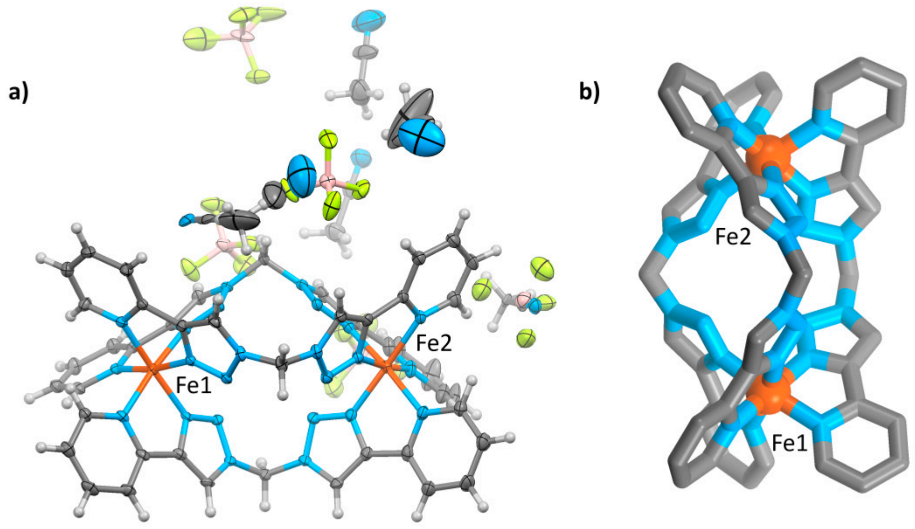

Figure S32. (**a**) Mercury ellipsoid plot of the asymmetric unit of $[Fe_2$**L1**$_3](BF_4)_4$·4.4CH_3CN, ellipsoids are shown at the 50% probability level; (**b**) Mercury tube representation of the mesocate. Colour

scheme: carbon = grey, hydrogen = white, nitrogen = blue, iron = orange, phosphorus = salmon, fluoride = yellow.

One of the tetrafluoroborate anions is disordered and modelled over two sites with the PART command, with 60:40 occupancy between the site containing B31b, F31b, F32b, F33b, and F34b and the site containing B31a, F31a, F32a, F33a, and F34a (**Error! Reference source not found.**S33). While the counterion is in its predominant site, centered on B31b, an acetonitrile solvent molecule is adjacent to it (N91b, C91b, C92), and the vacant anion site is occupied by another acetonitrile molecule (N71, C71, C72). When the counterion is centered on B31a, the first acetonitrile molecule swivels on the methyl carbon (C92) with C91a and N91a in the space vacated by the anion, and the other acetonitrile molecule (N71, C71, C72) is absent. Distances were fixed in the acetonitrile molecule containing N91, C91, C92 (both part a and b) using the DFIX command, and the ISOR command applied to C91b. Both B31a and B31b had the ISOR command applied to them, and boron-fluoride and fluoride-fluoride distances in both parts were fixed using the DFIX command. The ISOR command was also applied to N51 and C71, both within acetonitrile solvent molecules.

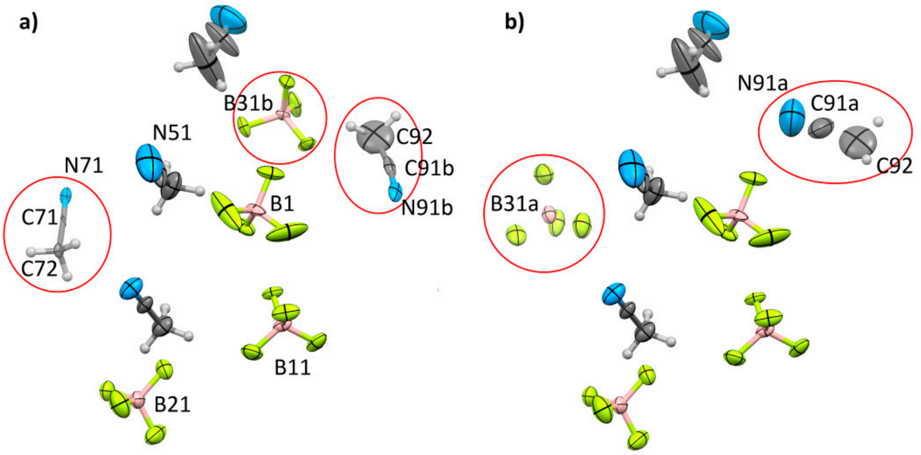

Figure S33. Mercury ellipsoid plot of the counterions and solvent molecules in the asymmetric unit of $[Fe_2\mathbf{L1}_3](BF_4)_4{\cdot}4.4CH_3CN$, showing (**a**) the 60% part; and (**b**) the 40% part. Ellipsoids are shown at the 50% probability level, Colour scheme: carbon = grey, hydrogen = white, nitrogen = blue, phosphorus = salmon, fluorine = yellow.

Table S2. Crystal data and structure refinement for $[Fe_2\mathbf{L1}_3](BF_4)_4{\cdot}4.4CH_3CN$.

Identification code	dp458b (CCDC 1491851)	
Empirical formula	$C_{53.80}H_{49.20}B_4F_{16}Fe_2N_{28.40}$	
Formula weight	1552.56	
Temperature	100.02(10) K	
Wavelength	1.54184 Å	
Crystal system	Triclinic	
Space group	$P\bar{1}$	
Unit cell dimensions	a = 12.5175(2) Å	a = 84.878(2)°
	b = 14.7657(3) Å	b = 78.200(2)°
	c = 18.2903(3) Å	g = 85.572(2)°
Volume	3289.91(10) Å^3	
Z	2	
Density (calculated)	1.567 Mg/m^3	
Absorption coefficient	4.494 mm^{-1}	
F(000)	1574	

Crystal size	0.3397 × 0.1771 × 0.1153 mm^3
Theta range for data collection	3.01° to 76.81°
Index ranges	−15 ≤ h ≤15, −18 ≤ k ≤18, −19 ≤ l ≤ 22
Reflections collected	52,255
Independent reflections	13738 [R(int) = 0.0357]
Completeness to theta = 76.81°	99.0%
Absorption correction	Gaussian
Max. and min. transmission	1.00000 and 0.68795
Refinement method	Full-matrix least-squares on F^2
Data/restraints/parameters	13,738/48/1021
Goodness-of-fit on F^2	1.189
Final R indices [I > 2sigma(I)]	R_1 = 0.0971, wR_2 = 0.2413
R indices (all data)	R_1 = 0.0977, wR_2 = 0.2423
Largest diff. peak and hole	2.406 and −0.797 e·$Å^{-3}$

2.2. $[Co_2\mathbf{L2}_3](NO_3)(Ce(NO_3)_6)(Ce(NO_3)_5(OH_2))\cdot 6CH_3NO_2$

Evaporation of a nitromethane solution of $[Co_2\mathbf{L3}_3](Ce(NO_3)_6)_2$ (pre-counterion exchange) gave yellow, rectangular crystals of $[Co_2\mathbf{L2}_3](NO_3)(Ce(NO_3)_6)(Ce(NO_3)_5(OH_2))\cdot 6CH_3NO_2$. X-ray data were collected at 100 K on an Agilent Technologies Supernova system using Cu Kα radiation with exposures over 1.0°, and data were treated using CrysAlisPro [6] software. The structure was solved using SHELXT [10], and weighted full-matrix-block refinement on F^2 was carried out using SHELXL-97 [8] running within the WinGX package [9]. All non-hydrogen atoms were refined anisotropically. Hydrogen atoms attached to carbons were placed in calculated positions and refined using a riding model. The structure was solved in the primitive triclinic space group $P\bar{1}$ and refined to an R_1 value of 21.3%. The asymmetric unit contains the dicobalt(III) mesocate, cerium(III) hexanitrate, cerium(III) pentanitratehydrate, and a nitrate as the counterions, as well as $6CH_3NO_2$ solvent molecules (**Error! Reference source not found.**S34).

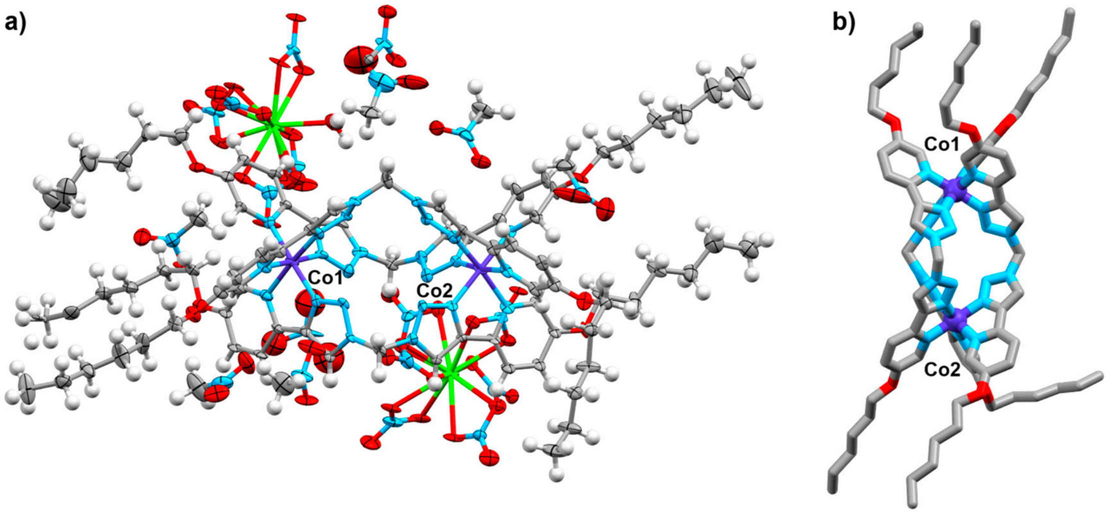

Figure S34. (**a**) Mercury ellipsoid plot of the asymmetric unit of $[Co_2\mathbf{L2}_3](NO_3)(Ce(NO_3)_6)(Ce(NO_3)_5(OH_2))\cdot 6CH_3NO_2$, ellipsoids are shown at the 50% probability level; (**b**) Mercury tube representation of the mesocate. Colour scheme: carbon = grey, hydrogen = white, nitrogen = blue, cobalt purple, cerium green.

The crystals were extremely fragile, and the one selected for data collection (the best after an extensive search) diffracted extremely poorly, only up to 0.96 Å. Furthermore, the crystal deteriorated

during the collection, and only partial data from the beginning of the collection was suitable for solving and refining the structure. Additionally, all other crystals, whether on the slide or within the mother liquor, decomposed during the time of the original data acquisition and did not diffract, preventing recollection of data. As such, the data collected are very low quality. Multiple atoms (>50%) were non-positive definite, and these were modelled using the ISOR and SIMU commands. There are multiple A and B alerts in the CIF file that relate to the high R factors and R_{int}, as well as high ADP max/min ratios. One of the methyl carbon atoms and associated hydrogen atoms are disordered and modelled over two sites with the PART command with 50:50 occupancy (C54a and C54b). In light of the poor data, it must be stressed that the structure can only be used to assess connectivity, which shows the $[M_2\mathbf{L}_3]^{6+}$ cationic structure as the expected mesocate. No bond angles, distances, or other structural data can be reliably extracted from the structure.

Table S3. Crystal data and structure refinement for $[Co_2\mathbf{L2}_3](NO_3)(Ce(NO_3)_6)(Ce(NO_3)_5(OH_2))\cdot 6CH_3NO_2$.

Identification code	RAVCo2 (CCDC 1505806)	
Empirical formula	$C_{88}H_{127}Ce_2Co_2N_{43}O_{57}$	
Formula weight	3097.42	
Temperature	115.45(10) K	
Wavelength	1.54184 Å	
Crystal system	Triclinic	
Space group	$P\bar{1}$	
Unit cell dimensions	a = 11.2314(3) Å	α = 77.082(3)°
	b = 23.3074(11) Å	β = 83.933(2)°
	c = 25.7949(5) Å	γ = 79.928(3)°
Volume	6464.5(4) Å^3	
Z	2	
Density (calculated)	1.591 Mg/m^3	
Absorption coefficient	8.259 mm^{-1}	
F(000)	3164	
Crystal size	0.3681 × 0.2813 × 0.1659 mm^3	
Theta range for data collection	3.524 to 73.832°.	
Index ranges	−12 ≤ h ≤ 13, −28 ≤ k ≤ 28, −31 ≤ l ≤ 32	
Reflections collected	96,717	
Independent reflections	25,486 [R(int) = 0.2497]	
Completeness to theta = 76.81°	99.9%	
Absorption correction	Gaussian	
Max. and min. transmission	1.00000 and 0.67340	
Refinement method	Full-matrix-block least-squares on F^2	
Data/restraints/parameters	25,486/618/1751	
Goodness-of-fit on F^2	1.048	
Final R indices [I > 2sigma(I)]	R_1 = 0.2135, wR_2 = 0.4756	
R indices (all data)	R_1 = 0.2456, wR_2 = 0.4938	
Largest diff. peak and hole	9.625 and −3.885 e·Å^{-3}	

3. Stability Studies Monitored by ^{1}H-NMR Spectroscopy

D_2O stability studies were conducted at ~0.5 mM; DMSO stability studies at ~0.7 mM; Cl^- stability study at ~0.6 mM in D_2O; histidine stability study at ~0.5 mM in D_2O.

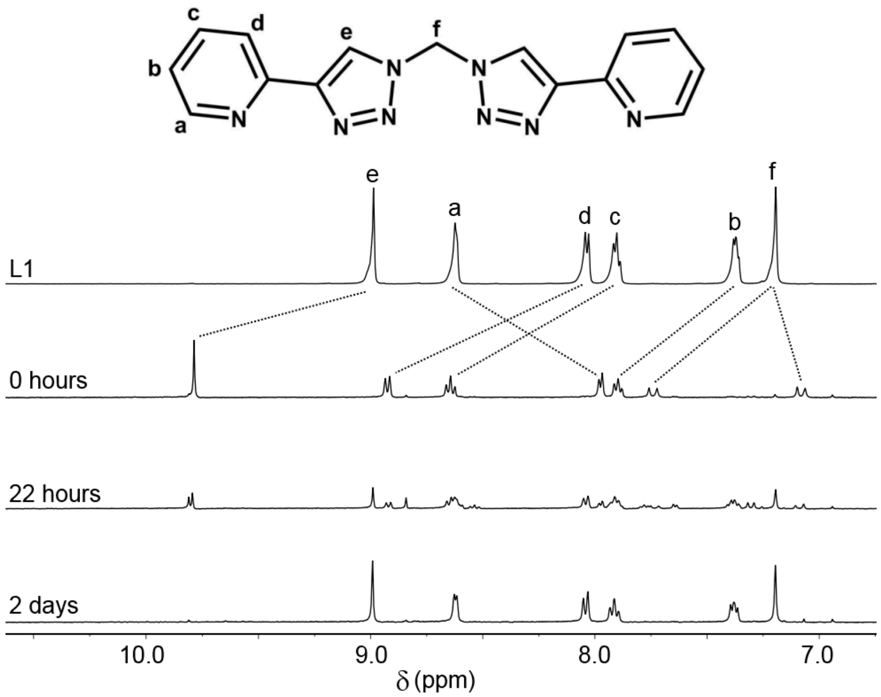

Figure S35. ^{1}H-NMR (400 MHz, DMSO, 298 K) stacked spectra of [Co_2**L1**$_3$]$(OTf)_6$ over 2 days in the light.

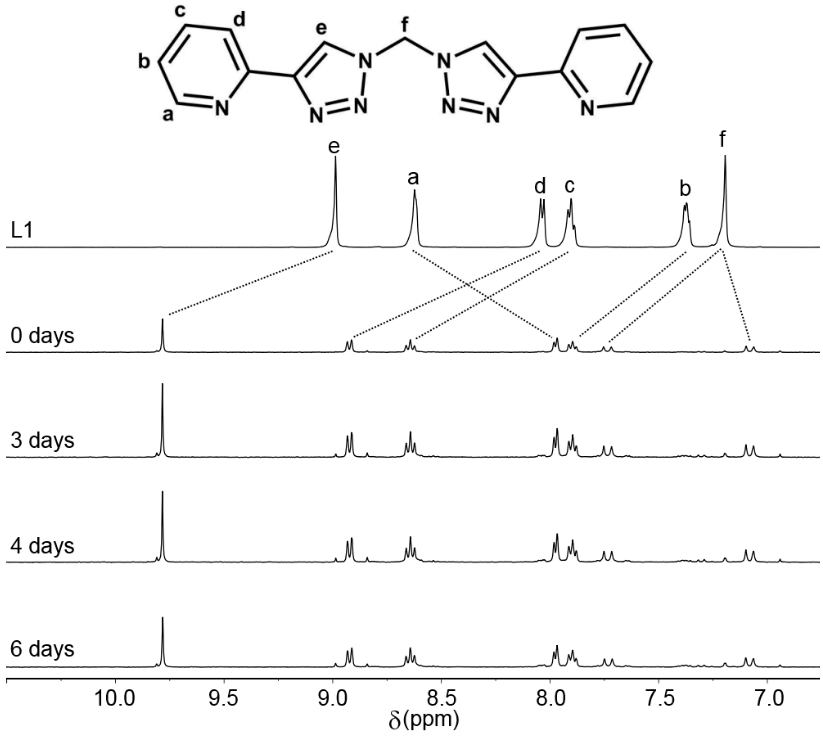

Figure S36. ^{1}H-NMR (400 MHz, DMSO, 298 K) stacked spectra of [Co_2**L1**$_3$]$(OTf)_6$ over 6 days in the dark.

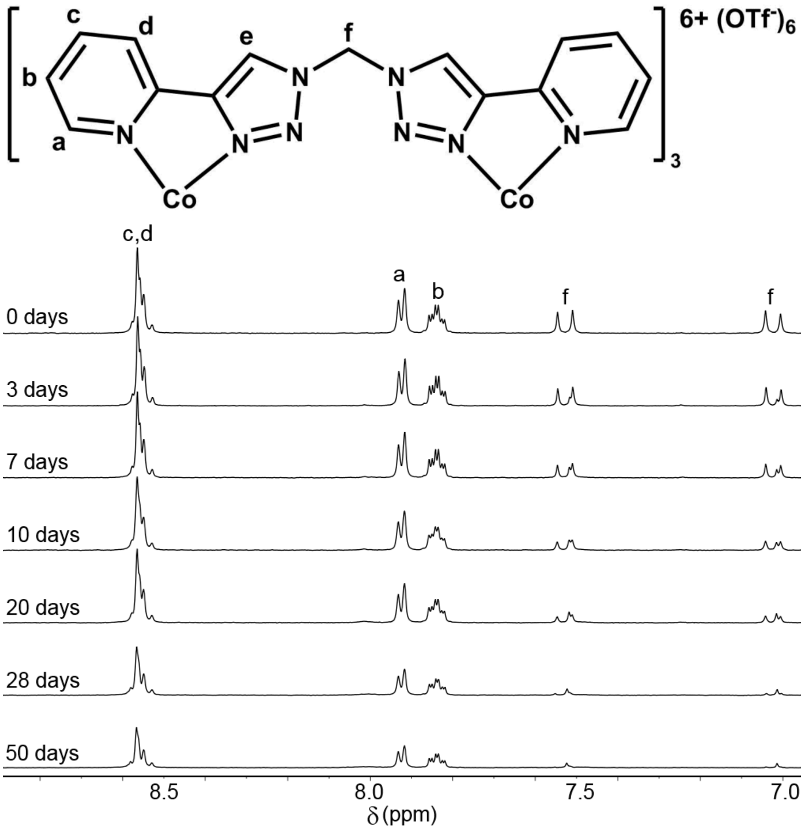

Figure S37. ^{1}H-NMR (400 MHz, D_2O, 298 K) stacked spectra of $[Co_2\mathbf{L1}_3](OTf)_6$ over 50 days. Note: **L1** is not soluble in D_2O. H_e is not observed, and H_f decreases in intensity due to deuterium exchange.

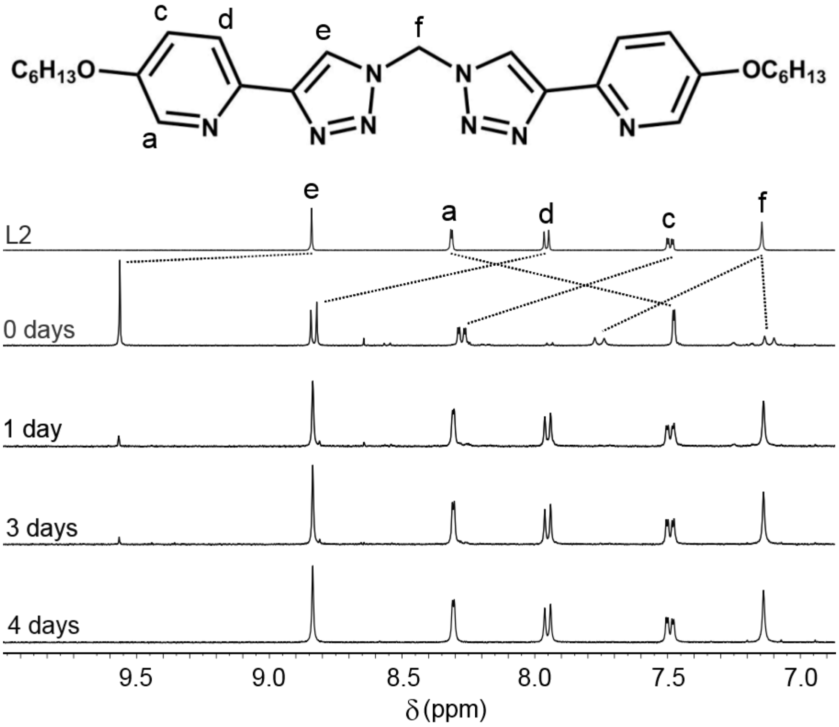

Figure S38. ^{1}H-NMR (400 MHz, DMSO, 298 K) stacked spectra of $[Co_2\mathbf{L2}_3](OTf)_6$ over 4 days in the light.

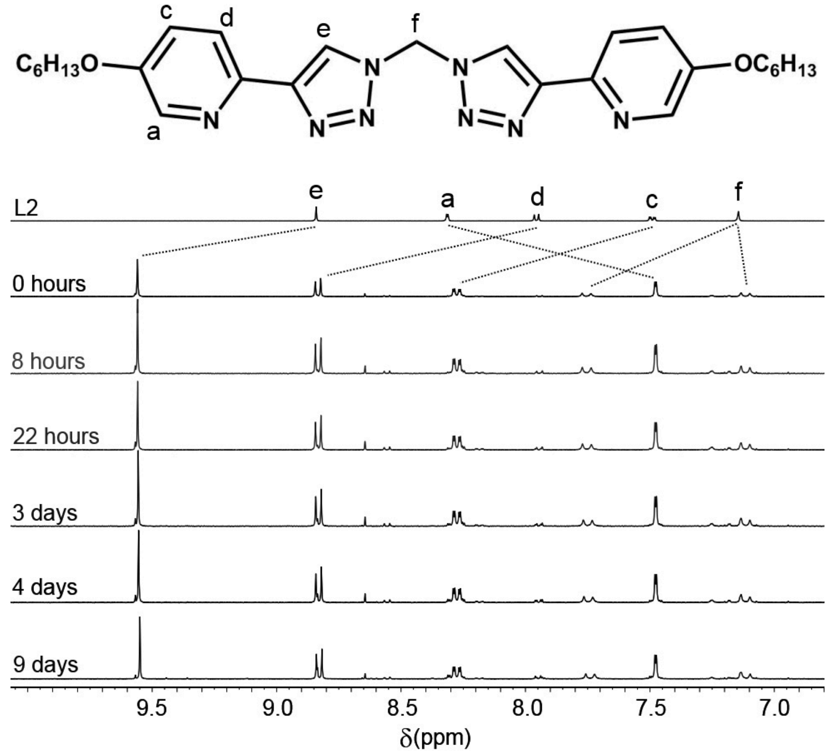

Figure S39. ^{1}H-NMR (400 MHz, DMSO, 298 K) stacked spectra of $[Co_2\mathbf{L2}_3](OTf)_6$ over 9 days in the dark.

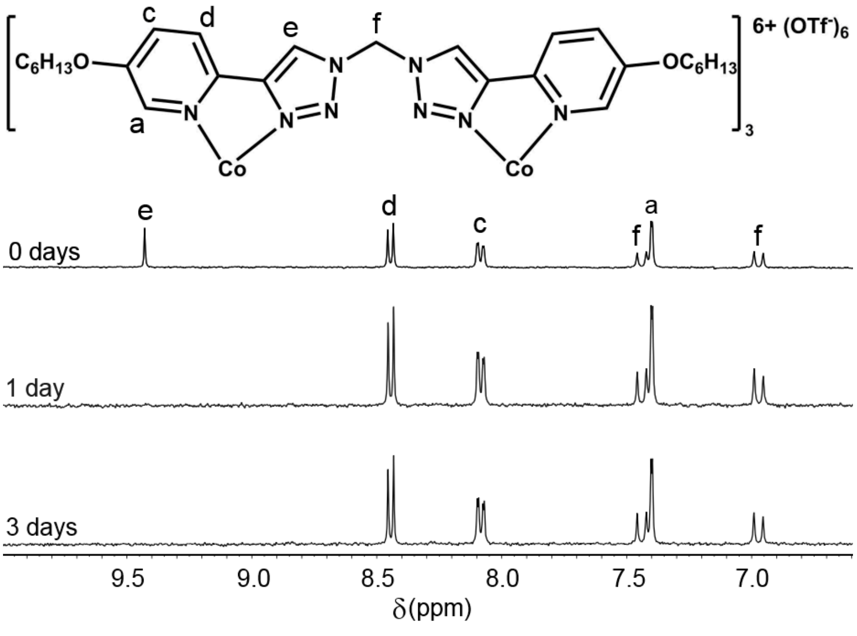

Figure S40. ^{1}H-NMR (400 MHz, D_2O, 298 K) stacked spectra of $[Co_2\mathbf{L2}_3](OTf)_6$ over 3 days. Note: **L2** is not soluble in D_2O. H_d disappears due to deuterium exchange.

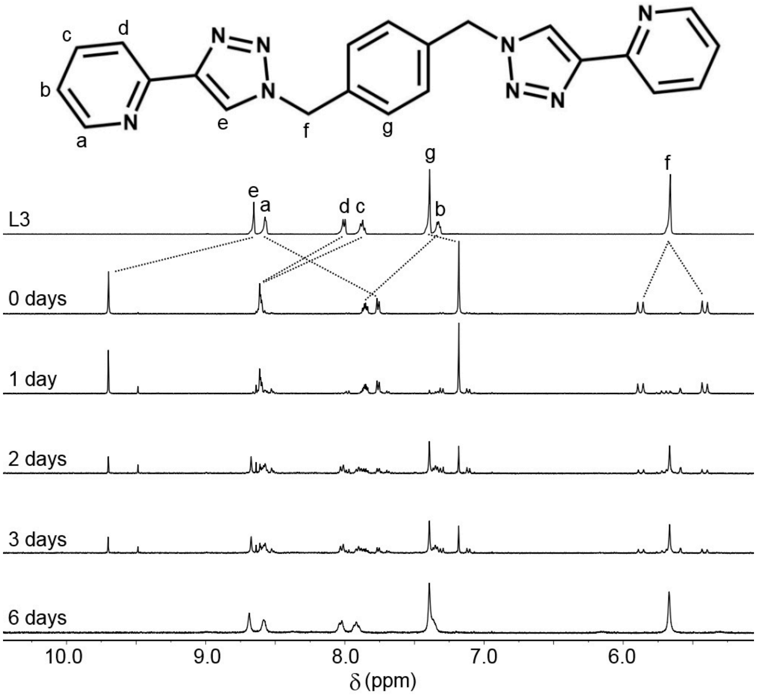

Figure S41. ^{1}H-NMR (400 MHz, DMSO, 298 K) stacked spectra of [Co_2**L3**$_3$]$(OTf)_6$ over 6 days in the light.

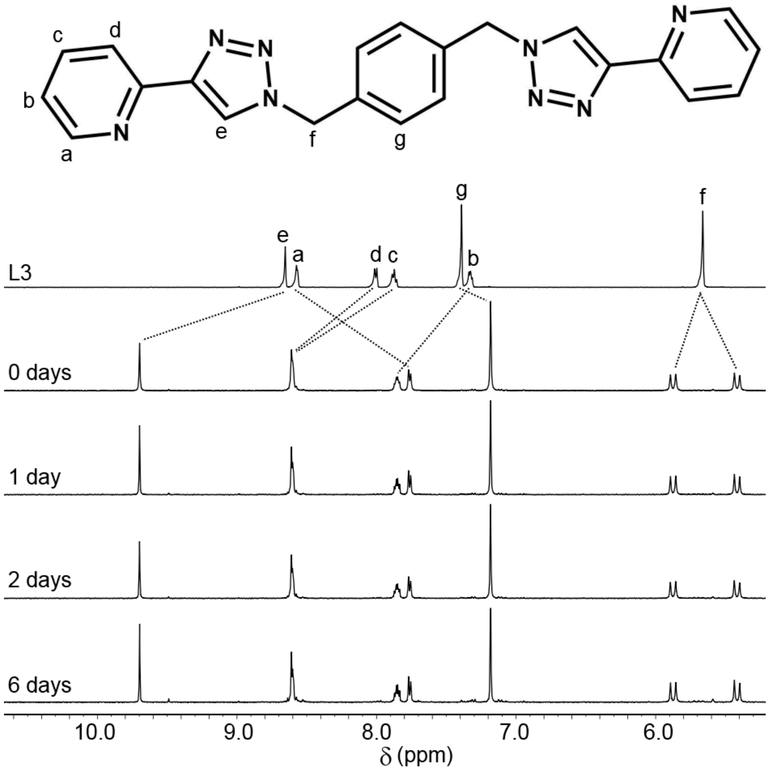

Figure S42. ^{1}H-NMR (400 MHz, DMSO, 298 K) stacked spectra of [Co_2**L3**$_3$]$(OTf)_6$ over 6 days in the dark.

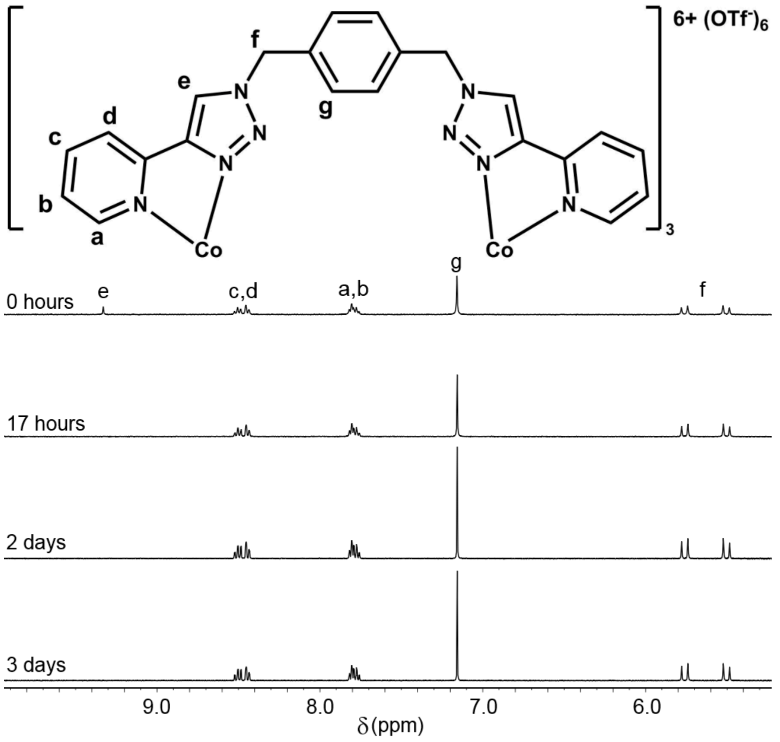

Figure S43. ^{1}H-NMR (400 MHz, D_2O, 298 K) stacked spectra of [Co_2**L3**$_3$]$(OTf)_6$ over 3 days. Note: **L3** is not soluble in D_2O. H_e disappears due to deuterium exchange.

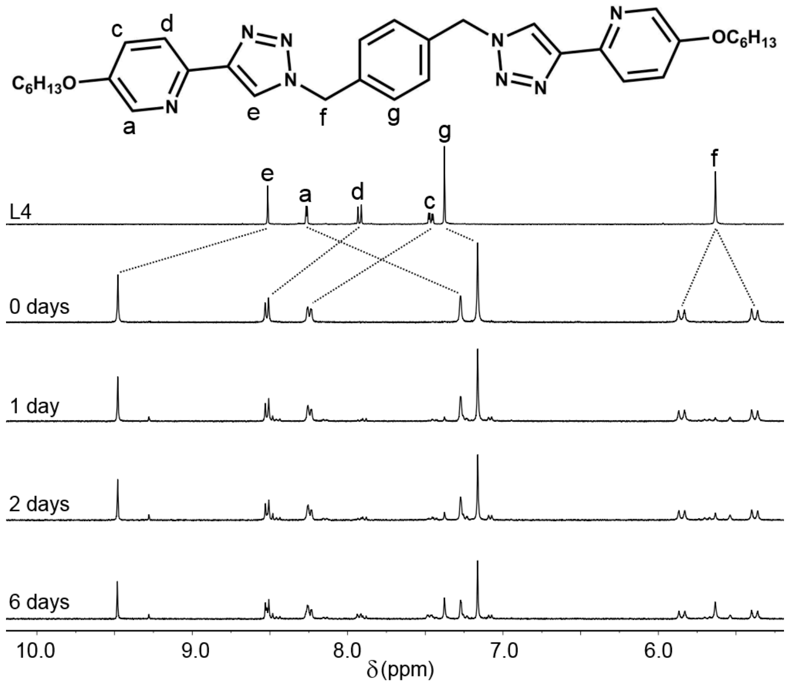

Figure S44. ^{1}H-NMR (400 MHz, DMSO, 298 K) stacked spectra of [Co_2**L4**$_3$]$(OTf)_6$ over 6 days in the light.

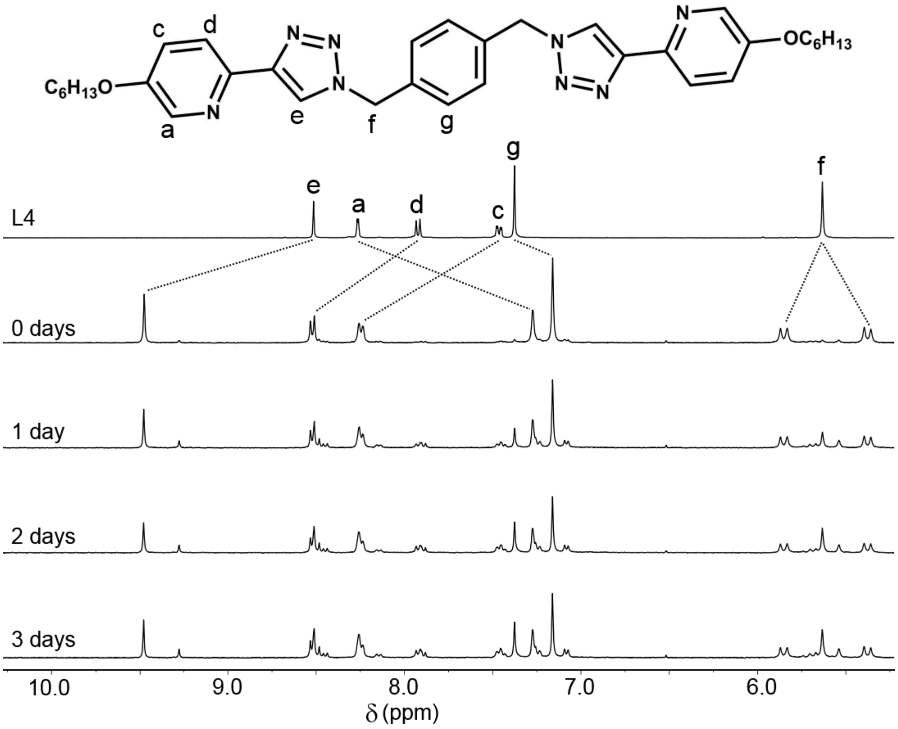

Figure S45. ^{1}H-NMR (400 MHz, DMSO, 298 K) stacked spectra of [Co_2**L4**$_3$]$(OTf)_6$ over 3 days in the dark.

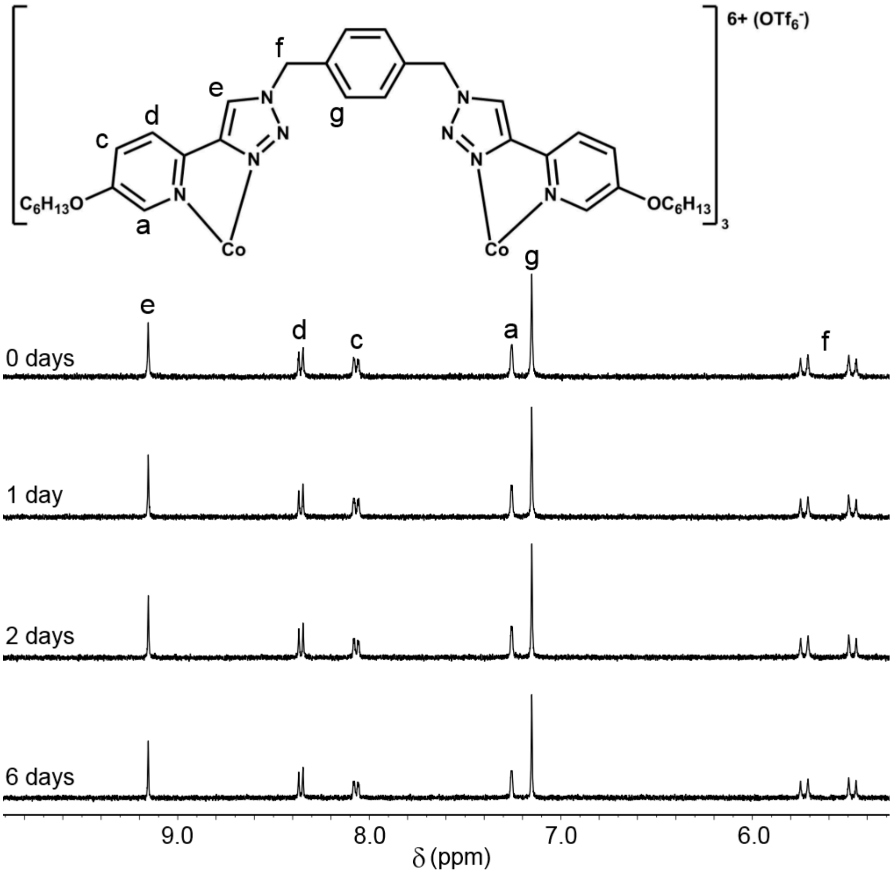

Figure S46. ^{1}H-NMR (400 MHz, D_2O, 298 K) stacked spectra of [Co_2**L4**$_3$]$(OTf)_6$ over 6 days. Note: **L4** is not soluble in D_2O.

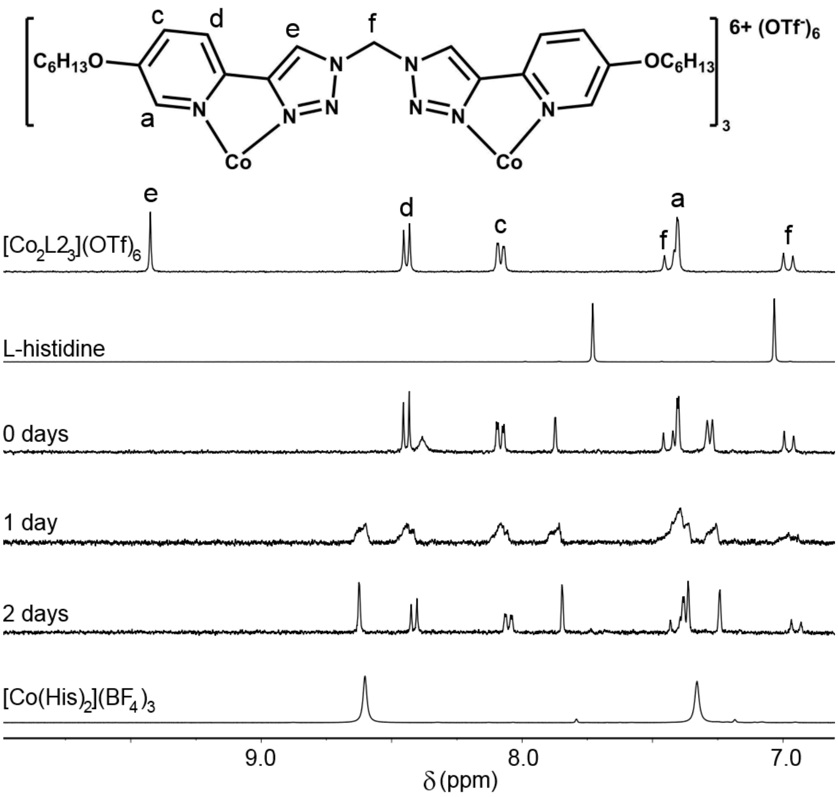

Figure S47. ^{1}H-NMR (400 MHz, D_2O, 298 K) stacked spectra of [Co_2**L2**$_3$](OTf)$_6$ in the presence of L-histidine over 2 days. Note: **L2** is not soluble in D_2O. H_d disappears due to deuterium exchange.

4. SPARTAN'16® Models of [Co_2L_3]$^{6+}$ Cylinders

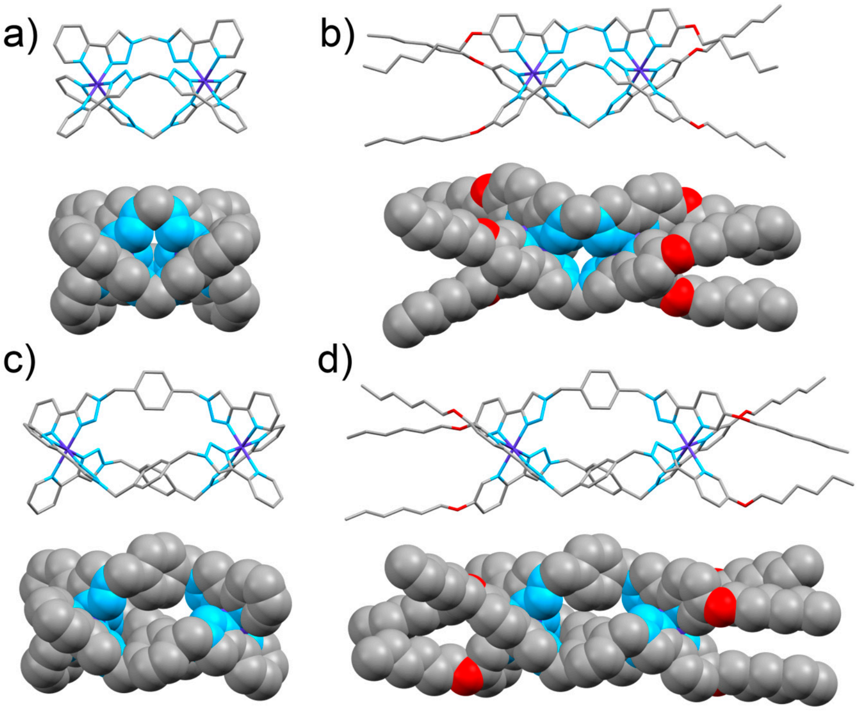

Figure S48. Energy optimised SPARTAN'16® models (MMFF molecular mechanics) of (**a**) [Co_2**L1**$_3$]$^{6+}$, capped sticks and space-filling models; (**b**) [Co_2**L2**$_3$]$^{6+}$; (**c**) [Co_2**L3**$_3$]$^{6+}$; (**d**) [Co_2**L4**$_3$]$^{6+}$. Co–Co distance for [Co_2**L1**$_3$]$^{6+}$ and [Co_2**L2**$_3$]$^{6+}$ = 7.04 Å. Co–Co distance for [Co_2**L3**$_3$]$^{6+}$ and [Co_2**L4**$_3$]$^{6+}$ = 11.53 Å.

5. Antibacterial Procedures

5.1. Kirby–Bauer Disk Diffusion Assays

Antibacterial activity was evaluated using the Kirby–Bauer disk diffusion assay. Each bacterial strain (*Escherichia coli* (ATC25922) and *Staphylococcus aureus* (ATC 25923)) was inoculated into a separate cation-adjusted Mueller–Hinton broth (MHB; BD, Auckland, New Zealand) and incubated at 35 ± 2 °C for a period of 24 h. The bacterial suspensions were subsequently adjusted to a 0.5 MacFarland opacity standard (1–2 × 10^8 colony forming units (CFU)/mL) and spread onto cation-adjusted Mueller–Hinton agar (BD, Auckland, New Zealand) plates before placing sterile paper disks (4 per plate, 6 mm diameter; BD, Auckland, New Zealand) equidistant on the plate. The water soluble cylinders were prepared by dissolving 1 mg of the compound in 1 mL of distilled water. The compounds (20 μL) were then introduced onto the disks. The plates were incubated for a period of 24 h at 35 ± 2 °C, after which the diameters of the zones of inhibition were measured. All through the course of these experiments gentamicin (10 μg discs; BD, Auckland, New Zealand) was used as a positive control, and the experiments were done in triplicate.

5.2. Minimum Inhibitory Concentration (MIC) (Broth Micro-Dilution Method)

The minimum inhibitory concentrations for water soluble compounds were determined by the broth micro-dilution method using 96-well U bottom tissue culture plates (Falcon, BD, Auckland, New Zealand). The initial 1–2 × 10^8 CFU/mL bacterial suspensions were diluted 1:100 with cation-adjusted Mueller–Hinton broth. Each well was inoculated with 100 μL of the bacterial cells and diluted with the same volume of the compounds (dissolved in broth and further filter sterilized using 0.20 μm pore size membrane filters (Sartorius Stedim Biotech, Germany), leading to a concentration range of 1024 μg/mL to 0.5 μg/mL and a bacterial concentration of 5 × 10^5 CFU/mL according to the CLSI guidelines [11]. Gentamicin (Gibco, Thermofisher Scientific NZ Ltd., Auckland, New Zealand) was used as the positive control and control and concentrations tested ranged from 16 to 0.125 μg/mL. Uninoculated broth was used as the sterility control. The inoculated broth devoid of the compound was used as the growth control. The plates were incubated at 35 ± 2 °C for 24 h. The lowest concentration at which the bacterial growth was inhibited was recorded as the MIC. The experiments were performed in triplicate.

References

1. Preston, D.; Tucker, R.A.J.; Garden, A.L.; Crowley, J.D. Heterometallic $[M_nPt_n(L)_{2n}]^{x+}$ Macrocycles from Dichloromethane-Derived Bis-2-pyridyl-1,2,3-triazole Ligands. *Inorg. Chem.* **2016**, *55*, 8928–8934.
2. Crowley, J.D.; Bandeen, P.H. A multicomponent CuAAC "click" approach to a library of hybrid polydentate 2-pyridyl-1,2,3-triazole ligands: New building blocks for the generation of metallosupramolecular architectures. *Dalton Trans.* **2010**, *39*, 612–623.
3. Chow, H.S.; Constable, E.C.; Housecroft, C.E.; Kulicke, K.J.; Tao, Y. When electron exchange is chemical exchange-assignment of 1H NMR spectra of paramagnetic cobalt(II)-2,2′:6′,2″-terpyridine complexes. *Dalton Trans.* **2005**, 236–237.
4. Kumar, S.V.; Lo, W.K.C.; Brooks, H.J.L.; Crowley, J.D. Synthesis, structure, stability and antimicrobial activity of a ruthenium(II) helicate derived from a bis-bidentate "click" pyridyl-1,2,3-triazole ligand. *Inorg. Chim. Acta* **2015**, *425*, 1–6.
5. Stephenson, M.D.; Hardie, M.J. Network structures with 2,2′-bipyridine-3,3′diol: A discrete Co(III) complex that forms a porous 3-D hydrogen bonded network, and Cu(II) coordination chains. *CrystEngComm* **2007**, *9*, 496–502.
6. *CrysAlisPro*; Agilent Technologies: Yarnton, UK, 2012.
7. Altomare, A.; Burla, M.C.; Camalli, M.; Cascarano, G.L.; Giacovazzo, C.; Guagliardi, A.; Moliterni, A.G.G.; Polidori, G.; Spagna, R. SIR97: A new tool for crystal structure determination and refinement. *J. Appl. Cryst.* **1999**, *32*, 115–119.
8. Sheldrick, G. A short history of SHELX. *Acta Cryst.* **2008**, *A64*, 112–122.
9. Farrugia, L. WinGX suite for small-molecule single-crystal crystallography. *J. Appl. Cryst.* **1999**, *32*, 837–838.

10. Sheldrick, G. SHELXT—Integrated space-group and crystal-structure determination. *Acta Cryst. A* **2015**, *71*, 3–8.
11. Central Laboratory Standards Institute. *Performance Standards for Antimicrobial Susceptibility Testing*, 22nd ed.; M100–222012; Clinical and Laboratory Standards Institute: Wayne, PA, USA, 2012.
